# Growth deficiency in a mouse model of Kabuki syndrome 2 bears mechanistic similarities to Kabuki syndrome 1

**DOI:** 10.1371/journal.pgen.1011310

**Published:** 2024-06-10

**Authors:** Christine W. Gao, WanYing Lin, Ryan C. Riddle, Sheetal Chopra, Jiyoung Kim, Leandros Boukas, Kasper D. Hansen, Hans T. Björnsson, Jill A. Fahrner

**Affiliations:** 1 Department of Genetic Medicine, Johns Hopkins University School of Medicine, Baltimore, Maryland, United States of America; 2 Department of Molecular Biology and Genetics, Johns Hopkins University School of Medicine, Baltimore, Maryland, United States of America; 3 Department of Orthopaedic Surgery, Johns Hopkins University School of Medicine, Baltimore, Maryland, United States of America; 4 Department of Orthopaedics, University of Maryland School of Medicine, Baltimore, Maryland, United States of America; 5 Research and Development Service, Baltimore Veterans Administration Medical Center, Baltimore, Maryland, United States of America; 6 Department of Biostatistics, Johns Hopkins University School of Public Health, Baltimore, Maryland, United States of America; 7 Department of Biomedical Engineering, Johns Hopkins University School of Medicine, Baltimore, Maryland, United States of America; 8 Department of Pediatrics, Johns Hopkins University School of Medicine, Baltimore, Maryland, United States of America; 9 Faculty of Medicine, University of Iceland, Reykjavík, Iceland; 10 Landspítali University Hospital, Reykjavík, Iceland; Broad Institute, UNITED STATES

## Abstract

Growth deficiency is a characteristic feature of both Kabuki syndrome 1 (KS1) and Kabuki syndrome 2 (KS2), Mendelian disorders of the epigenetic machinery with similar phenotypes but distinct genetic etiologies. We previously described skeletal growth deficiency in a mouse model of KS1 and further established that a *Kmt2d*^*-/-*^ chondrocyte model of KS1 exhibits precocious differentiation. Here we characterized growth deficiency in a mouse model of KS2, *Kdm6a*^*tm1d/+*^. We show that *Kdm6a*^*tm1d/+*^ mice have decreased femur and tibia length compared to controls and exhibit abnormalities in cortical and trabecular bone structure. *Kdm6a*^*tm1d/+*^ growth plates are also shorter, due to decreases in hypertrophic chondrocyte size and hypertrophic zone height. Given these disturbances in the growth plate, we generated *Kdm6a*^*-/-*^ chondrogenic cell lines. Similar to our prior *in vitro* model of KS1, we found that *Kdm6a*^*-/-*^ cells undergo premature, enhanced differentiation towards chondrocytes compared to *Kdm6a*^*+/+*^ controls. RNA-seq showed that *Kdm6a*^*-/-*^ cells have a distinct transcriptomic profile that indicates dysregulation of cartilage development. Finally, we performed RNA-seq simultaneously on *Kmt2d*^*-/-*^, *Kdm6a*^*-/-*^, and control lines at Days 7 and 14 of differentiation. This revealed surprising resemblance in gene expression between *Kmt2d*^*-/-*^ and *Kdm6a*^*-/-*^ at both time points and indicates that the similarity in phenotype between KS1 and KS2 also exists at the transcriptional level.

## Author summary

Mendelian disorders of the epigenetic machinery (MDEMs) are an individually rare but collectively common group of disorders that disrupt neurodevelopment and growth. We have focused on abnormal skeletal growth. MDEMs are caused by disruption of genes encoding components of the epigenetic machinery, which ultimately lead to broadly altered cellular gene expression patterns. These disorders can therefore inform our understanding of epigenomic and transcriptomic mechanisms underlying normal and aberrant skeletal growth. We previously elucidated novel mechanisms contributing to overgrowth in Weaver syndrome and to growth deficiency in Kabuki syndrome 1. Here, we elucidate the skeletal growth deficiency phenotype in a mouse model of Kabuki syndrome 2, which resembles some aspects in Kabuki syndrome 1 mice and the human disease. We observe shortened bones with altered structure, involving reduced growth plate height and chondrocyte cell size in our mice. Moreover, we establish precocious differentiation of chondrocytes as a cellular mechanism and observe a disproportionate upregulation of chondrogenic genes. Remarkably, gene expression changes in Kabuki syndrome 2 chondrocytes substantially overlap with Kabuki syndrome 1. This suggests shared disease mechanisms between the disorders at the level of gene expression, which may facilitate targeting both disorders with a single therapy in the future.

## Introduction

Kabuki syndromes 1 and 2 (respectively, KS1 and KS2; MIM 147920 and 300867) are Mendelian disorders of the epigenetic machinery (MDEMs) [1] with shared features of postnatal growth deficiency including short stature and microcephaly, intellectual disability/developmental delay, hypotonia, immune dysfunction, and strikingly similar facial features [2]. Patients with either type of Kabuki syndrome are recognizable by their highly arched eyebrows (notched in some cases), elongated palpebral fissures with eversion of the lateral third of the lower eyelid, shortened columella, prominent ears, and persistent fingertip pads. Despite the clinical similarities between KS1 and KS2, their genetic etiologies are distinct.

KS1 accounts for up to 75% of Kabuki syndrome cases [3], and originates from heterozygous, typically *de novo*, pathogenic variants in *KMT2D* [4], which encodes a histone lysine methyltransferase that mono-, di-, and tri-methylates H3K4 [5,6]. KMT2D acts at both enhancers and promoters to regulate gene expression. In general, H3K4me1 placed at enhancers and H3K4me3 at promoters are observed at transcriptionally active regions [7]. KS2 is comparatively rare, representing 5–8% of Kabuki syndrome [8]. Its causative gene, *KDM6A* [9,10], encodes a H3K27 demethylase that preferentially acts upon H3K27me3 and H3K27me2 [11,12]. Both modifications are associated with silenced regions of the genome, with H3K27me3 marking transcriptionally repressed promoters, and H3K27me2 coating non-transcribed, intergenic swaths [13,14]. *KDM6A* is on the X chromosome, although it is known to escape X inactivation [15]. A *KDM6A* homologue, *UTY*, exists on the Y chromosome, but is catalytically inactive [12,16]. This imbalance in gene dosage is thus hypothesized to be responsible for the more severe phenotype seen in males with KS2 as opposed to females. Among individuals with KS2, males have significantly lower birth length and age-adjusted height than females, and a significantly lower percentage of male patients exhibit independent walking and developed speech [17].

Given the distinct origins of KS1 and KS2, this pair of syndromes provides an interesting study for how divergent epigenetic mechanisms may converge upon similar phenotypes. Fahrner and Björnsson previously proposed the ‘Balance Hypothesis’, such that writers and erasers of epigenetic marks exist in a balance to maintain a normal chromatin state [1]. Losing a writer of an activating mark (KMT2D; H3K4 methylation) or losing the eraser of a silencing mark (KDM6A; H3K27 methylation) are both predicted to lead to a closed chromatin state and corresponding transcriptional repression at target genes. Interestingly, chromatin accessibility profiling via ATAC-seq of B cells derived from KS1 and KS2 mouse models showed the converse effect of increased accessibility for both disorders specifically at promoters; such bias was not seen in distal regulatory elements [18]. This raised the possibility of indirect effects dominating at the chromatin level–for example, loss of KMT2D or KDM6A may lead to transcriptional silencing of repressors of chromatin accessibility, leading to a paradoxical gain in accessibility. At the transcriptional level, B cells from *Kmt2d*^*βgeo/+*^ and *Kdm6a*^*tm1d/+*^ mice bear considerable overlap in differentially expressed genes when compared to wild-type littermates, suggesting that the same pathways are perturbed in the immune cells of KS1 and KS2. To our knowledge, comparisons between the two disorders have not been made in other affected tissue types.

Growth deficiency is a central feature of both KS1 and KS2. The heights of children with KS1 are on average ≤2 standard deviations for age-adjusted mean [19]. KS2-specific growth curves are unavailable due to the rarity of the disorder, but growth deficiency is known to be global and proportionate, with some children also afflicted with microcephaly [3,20,21]. We previously used *Kmt2d*^*βgeo/+*^ mice as a model for growth abnormalities in KS1, and showed that disrupted endochondral ossification due to precocious differentiation of chondrocytes contributes towards growth deficiency in KS1 [22]. *Kmt2d*^*βgeo/+*^ mice had shortened femurs and tibias, and abnormally increased tibial growth plate heights. Cartilaginous growth plates, which expand upon proliferation and hypertrophy of chondrocytes, serve as scaffolds for bone matrix deposition by invading osteoblasts, and are critical to longitudinal bone growth. The finding in *Kmt2d*^*βgeo/+*^ mice of shortened bones in the setting of taller growth plates thus appeared counterintuitive. However, we also saw paradoxically accelerated differentiation and altered transcriptional profile of *Kmt2d*^*-/-*^ chondrocytes *in vitro*. Our data therefore support the idea that growth deficiency in KS1 stems from perturbations to chondrocytes in the growth plates.

Here, we expand our focus to growth deficiency in KS2. We leverage the *Kdm6a*^*tm1d/+*^ mouse model of KS2 to characterize skeletal and growth plate abnormalities in this related disorder. In light of our previous findings in KS1, we create *Kdm6a*^*-/-*^ chondrogenic cell lines to investigate chondrocyte differentiation in KS2. We perform RNA-seq of both *Kdm6a*^*-/-*^ and *Kmt2d*^*-/-*^ cells to compare gene expression at different time points and across the two disorders. Our work supports a convergent pattern of gene expression and a common mechanism involving precocious chondrocyte differentiation that leads to growth deficiency in both KS1 and KS2.

## Results

### *Kdm6a*^*tm1d/+*^ mice exhibit growth deficiency

**Fig 1:**
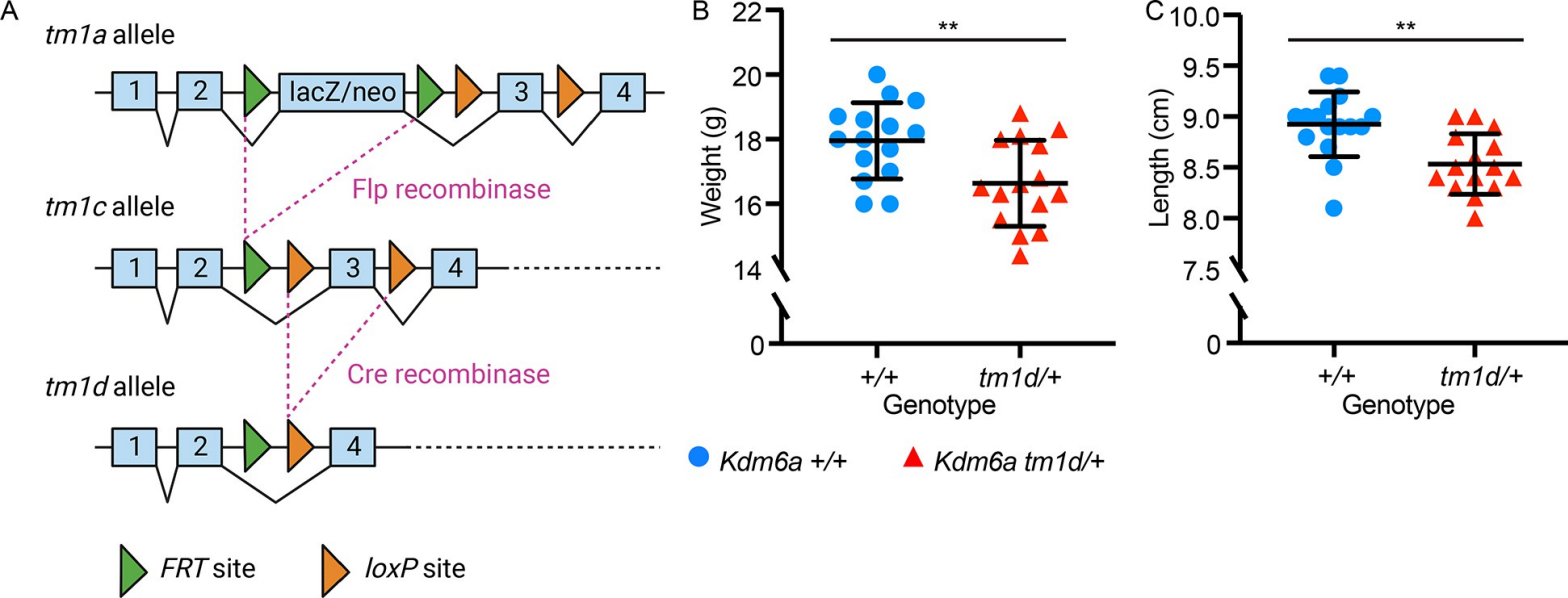
*Kdm6a*^*tm1d/+*^ mice exhibit generalized growth deficiency. (A) Schematic of the targeted *Kdm6a* allele as designed by the International Knockout Mouse Consortium, showing *FRT* sites as green arrows and *loxP* sites as orange arrows. Generation of the *tm1d* knockout allele requires excision of exon 3. Created using BioRender.com. Compared to *Kdm6a*^*+/+*^ littermates, *Kdm6a*^*tm1d/+*^ mice have significantly decreased (B) body weight (*Kdm6a*^*+/+*^ n = 15; *Kdm6a*^*tm1d/+*^ n = 15) and (C) body length (*Kdm6a*^*+/+*^ n = 16; *Kdm6a*^*tm1d/+*^ n = 15). Measurements obtained at 8 weeks of age. Blue circles: *Kdm6a*^*+/+*^, red triangles: *Kdm6a*^*tm1d/+*^. **p < 0.01, two-tailed unpaired Student’s t-test. All error bars represent mean ± 1 SD.

We utilized a previously described mouse model of KS2, *Kdm6a*^*tm1d(EUCOMM)Wtsi*^ [18], generated using the ‘knockout-first’ targeted construct created by the International Knockout Mouse Consortium (IKMC) [23] and referred to as *Kdm6a*^*tm1d/+*^ henceforth. The *tm1d* allele results from Cre-mediated excision of *Kdm6a* exon 3, which leads to a frameshift and premature termination codon (PTC) in exon 6 of 29 (Fig 1A). Female heterozygous *Kdm6a*^*tm1d/+*^ mice were viable and born at term. However, they experienced an increased rate of perinatal lethality, and survivors had significantly decreased body weight and length at 8 weeks compared to female *Kdm6a*^*+/+*^ littermates (p = 0.007 and p = 0.001, respectively) (Fig 1B and 1C). Male pups hemizygous for *Kdm6a* were previously described to succumb perinatally [24], and we likewise observed complete perinatal lethality in *Kdm6a*^*tm1d/y*^ pups. For subsequent experiments, therefore, we focused on heterozygous female mice.

**Fig 2:**
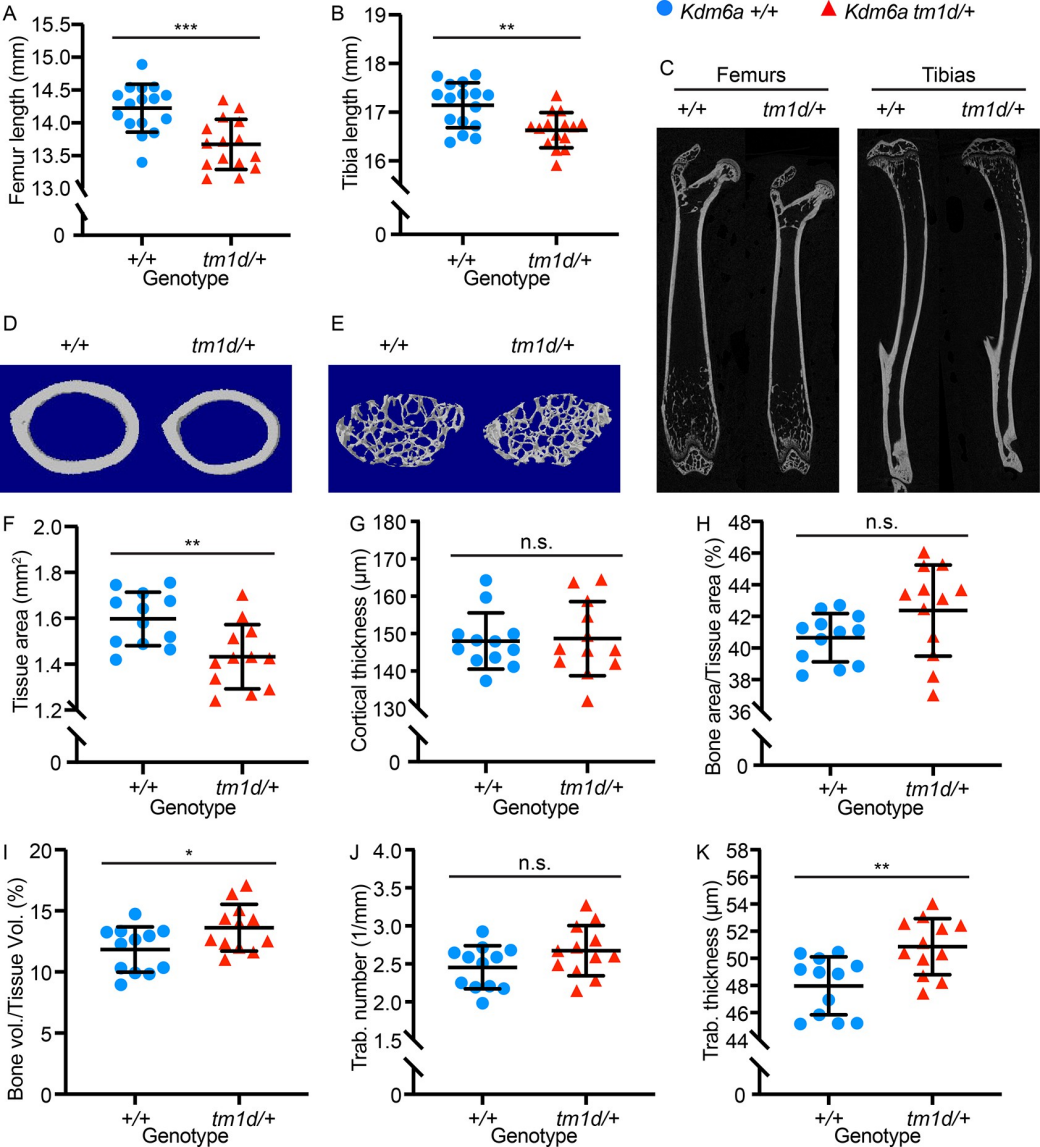
*Kdm6a*^*tm1d/+*^ mice have shortened bones and altered skeletal parameters. *Kdm6a*^*tm1d/+*^ mice have significantly shorter (A) femurs and (B) tibias compared to *Kdm6a*^*+/+*^ littermates (*Kdm6a*^*+/+*^ n = 16; *Kdm6a*^*tm1d/+*^ n = 15), which is also shown in (C) representative longitudinal micro-CT images of femurs and tibias from *Kdm6a*^*tm1d/+*^ and *Kdm6a*^*+/+*^. Representative transverse cross-sectional micro-CT reconstructions are displayed for femoral (D) cortical bone and (E) trabecular bone. *Kdm6a*^*tm1d/+*^ mice have decreased (F) femoral cross-sectional tissue area. However, there is no significant difference in (G) cortical thickness or (H) bone area/tissue area percentage, despite *Kdm6a*^*tm1d/+*^ mice displaying a slight upward trend in the latter. (I) Trabecular volume is increased in *Kdm6a*^*tm1d/+*^ mice. While (J) trabecular number does not differ significantly, (K) trabecular thickness is increased in *Kdm6a*^*tm1d/+*^ mice. For (F) through (K), *Kdm6a*^*+/+*^ n = 12; *Kdm6a*^*tm1d/+*^ n = 12. Blue circles: *Kdm6a*^*+/+*^, red triangles: *Kdm6a*^*tm1d/+*^. *p < 0.05, **p < 0.01, ***p < 0.001, two-tailed unpaired Student’s t-test. All error bars represent mean ± 1 SD. n.s., non-significant.

To assess growth abnormalities in *Kdm6a*^*tm1d/+*^ mice, we first performed skeletal profiling. *Kdm6a*^*tm1d/+*^ mice had shorter femurs and tibias than *Kdm6a*^*+/+*^ littermates (p = 0.0003 and p = 0.001, respectively) at 8 weeks of age (Fig 2A and 2B). Micro-computed tomography (micro-CT) performed concurrently revealed long bone structural differences in both cortical and trabecular parameters (Fig 2C–2E). *Kdm6a*^*tm1d/+*^ mice had decreased tissue area in the femoral mid-diaphysis (p = 0.005) (Fig 2D and 2F), which was significant even after normalization for femur length (p = 0.033) (S1 Fig). Despite the decreased tissue area, cortical thickness was unaffected in *Kdm6a*^*tm1d/+*^ mice (Fig 2G), leading to a trend towards a higher bone area-to-tissue area percentage (p = 0.081) (Fig 2H). Trabecular volume was increased in *Kdm6a*^*tm1d/+*^ mice (p = 0.030) (Fig 2E and 2I), secondary to a trend towards higher trabecular number (p = 0.097) (Fig 2E and 2J) and strikingly increased trabecular thickness in *Kdm6a*^*tm1d/+*^ mice (p = 0.003) (Fig 2E and 2K). These changes suggest disturbances to long bone development due to the heterozygous loss of *Kdm6a*.

### Growth plate structure is disrupted in *Kdm6a*^*tm1d/+*^ mice

**Fig 3:**
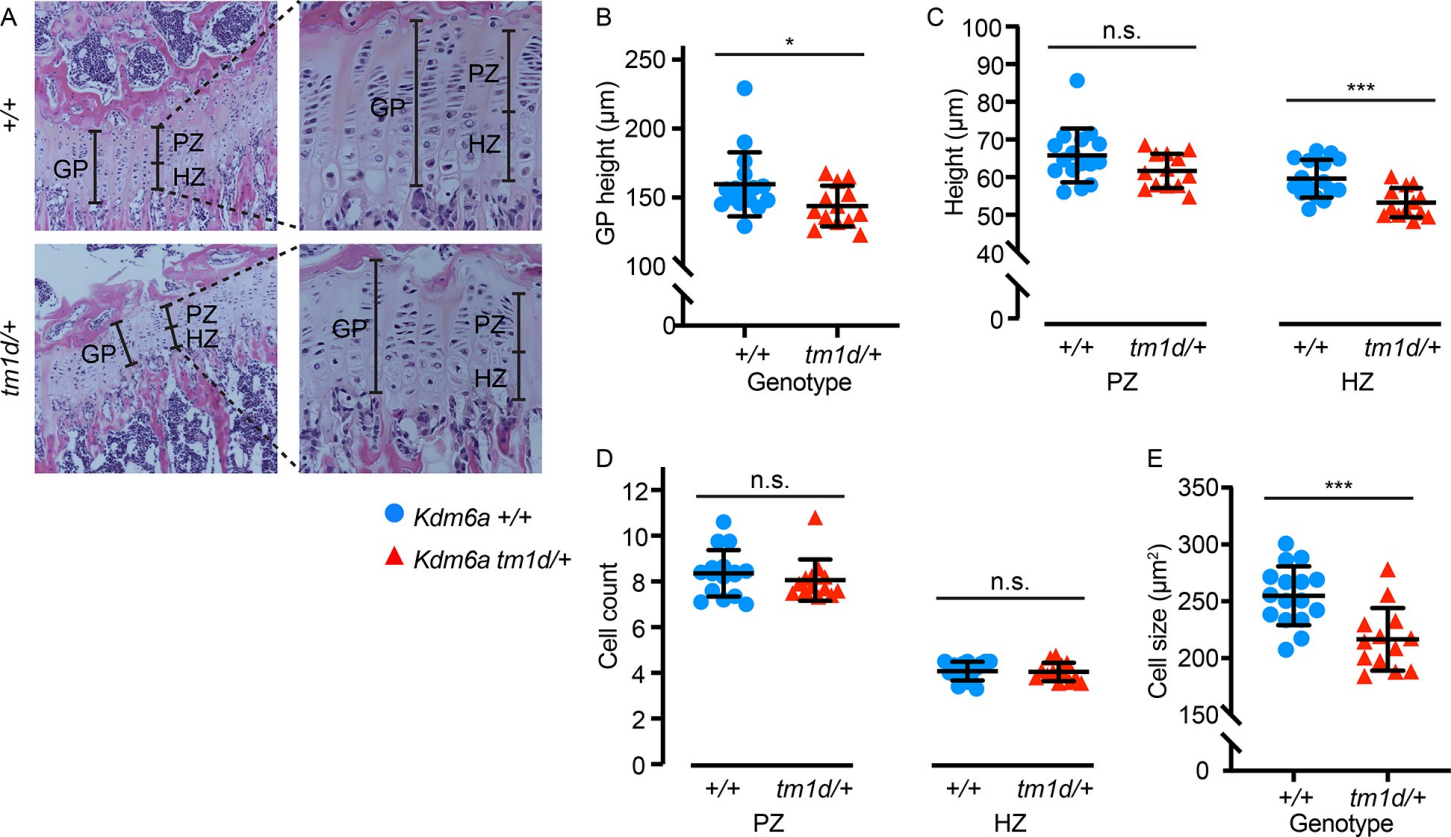
Growth plates have altered structure in *Kdm6a*^*tm1d/+*^ mice. (A) Hematoxylin and eosin staining of *Kdm6a*^*tm1d/+*^ and *Kdm6a*^*+/+*^ tibial growth plates. Left panels: 10x magnification. Insets: 40x magnification. (B) Growth plate height is decreased in *Kdm6a*^*tm1d/+*^ mice. This can be attributed to (C) shorter hypertrophic zones (right panel) in *Kdm6a*^*tm1d/+*^ mice, although proliferative zone heights do not differ (left panel). (D) Cell counts do not differ in either the proliferative (left) or hypertrophic (right) zones, but (E) cross-sectional cell size in the hypertrophic zone is smaller in *Kdm6a*^*tm1d/+*^ growth plates. *Kdm6a*^*+/+*^ n = 16; *Kdm6a*^*tm1d/+*^ n = 13. Blue circles: *Kdm6a*^*+/+*^, red triangles: *Kdm6a*^*tm1d/+*^. *p < 0.05, ***p < 0.001, two-tailed unpaired Student’s t-test. All error bars represent mean ± 1 SD. n.s., non-significant. GP, growth plate. PZ, proliferative zone. HZ, hypertrophic zone.

We examined whether perturbed bone development may stem from defects in the growth plate by performing hematoxylin and eosin (H&E) staining of proximal tibial growth plates sectioned in the longitudinal plane at 8 weeks of age (Fig 3A). *Kdm6a*^*tm1d/+*^ mice had an overall decrease in growth plate height (p = 0.042) (Fig 3A and 3B). This was caused by a significant shortening of the hypertrophic zone in *Kdm6a*^*tm1d/+*^ mice compared to wild-type (p = 0.0008), although the proliferative zone did not differ from wild-type littermates (Fig 3A and 3C). Cell counts in both the proliferative and hypertrophic zones were unchanged between the genotypes (Fig 3D); similarly, there was no difference in cell proliferation by EdU incorporation assay (S2 Fig). Instead, hypertrophic chondrocyte cell size, as measured by cross-sectional cell area in the longitudinal plane, was decreased in *Kdm6a*^*tm1d/+*^ mice (p = 0.0006) (Fig 3E). Chondrocyte hypertrophy is the main driver of longitudinal bone growth [25]. These changes in the growth plate, particularly in the hypertrophic zone, suggest that an abnormality of chondrocyte function is involved in the pathogenesis of growth deficiency in KS2.

### *Kdm6a*^*-/-*^ chondrogenic cell lines exhibit precocious differentiation at both phenotypic and transcriptomic levels

**Fig 4:**
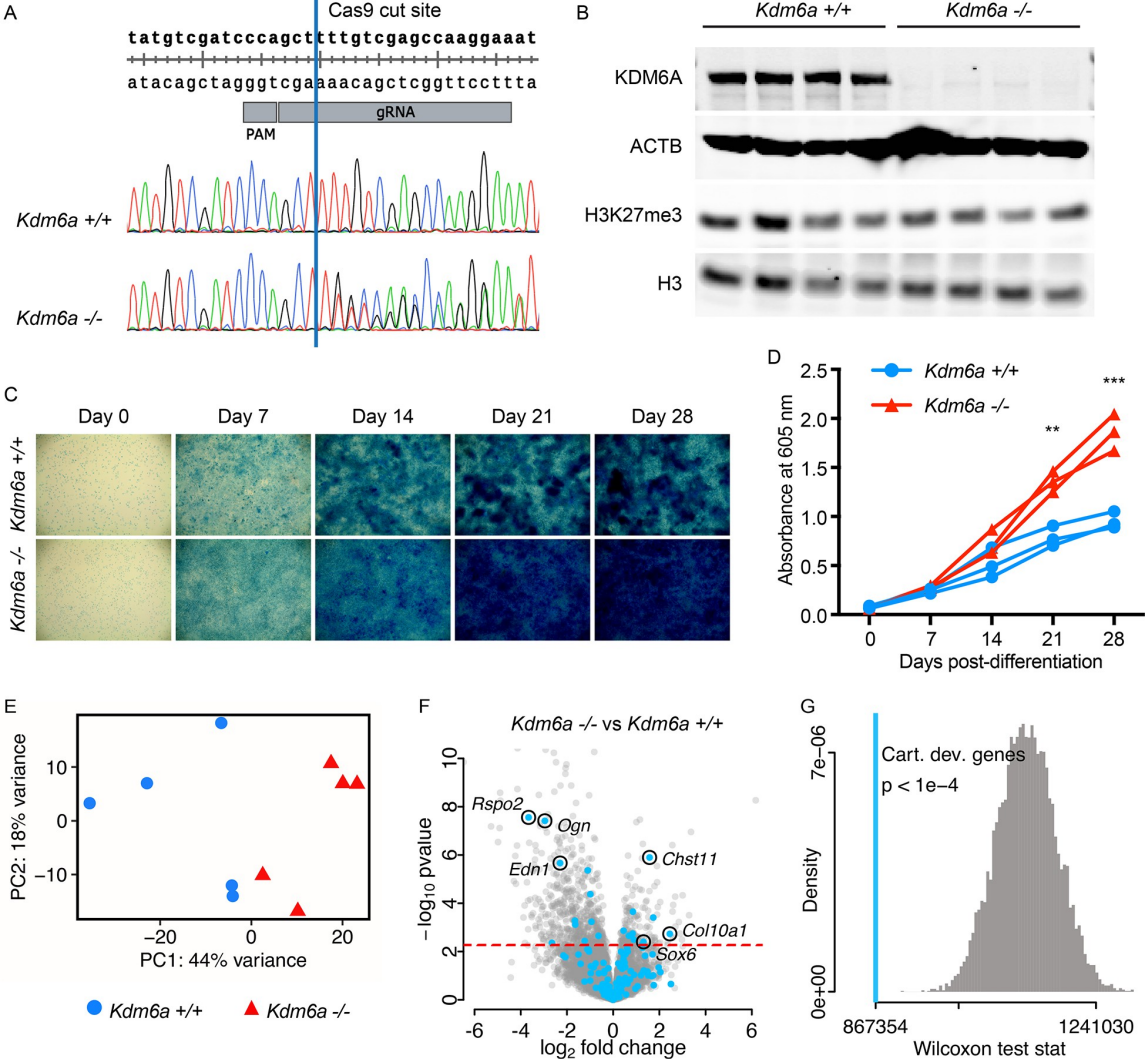
*Kdm6a*^*-/-*^ cells exhibit dysregulated chondrogenesis. (A) CRISPR-Cas9 gRNA target site for generating *Kdm6a*^*-/-*^ cell lines. Representative chromatograms from an unedited *Kdm6a*^*+/+*^ clone and a compound heterozygous *Kdm6a*^*-/-*^ clone, which shows double peaks following the intended Cas9 cut site (blue line). (B) *Kdm6a*^*-/-*^ cells do not express KDM6A protein, and H3K27me3 levels are unaltered from *Kdm6a*^*+/+*^ control lines. (C) Representative Alcian blue staining images (4x magnification) from a 28-day time course of *Kdm6a*^*-/-*^ and *Kdm6a*^*+/+*^ cell lines. (D) *Kdm6a*^*-/-*^ cells lines have increased uptake of Alcian blue stain. Each line represents a single technical replicate, comprising the average of 3–4 biological replicates. (E) Principal component analysis of *Kdm6a*^*-/-*^ and *Kdm6a*^*+/+*^ RNA-seq samples collected at Day 14 of differentiation (*Kdm6a*^*+/+*^ n = 5; *Kdm6a*^*-/-*^ n = 5). Blue circles: *Kdm6a*^*+/+*^, red triangles: *Kdm6a*^*-/-*^. (F) Volcano plot for *Kdm6a*^*-/-*^ versus *Kdm6a*^*+/+*^ samples, with MGI cartilage development genes highlighted in light blue. False discovery rate (FDR) = 0.1 (red dashed line), which corresponds to a p-value of 0.0055. (G) Wilcoxon rank sum test statistic for MGI cartilage development genes (light blue line, p < 1e-4) and simulated distribution of test statistics (gray). **p < 0.01, ***p < 0.001, two-tailed unpaired Student’s t-test. Cart. dev. genes, cartilage development genes.

To create a tractable *in vitro* model for chondrocyte differentiation and development in KS2, we chose to use ATDC5, a well-characterized female murine teratocarcinoma cell line that is known to undergo chondrogenesis when induced to differentiate [26–28]. CRISPR-Cas9 editing was used to target *Kdm6a* exon 6, introducing indels by non-homologous end joining (NHEJ) (Fig 4A). We isolated clones that remained unedited (*Kdm6a*^*+/+*^) as controls, alongside clones bearing compound heterozygous frameshift mutations (*Kdm6a*^*-/-*^). All such frameshift mutations led to premature termination codons (PTCs) upstream of the catalytic Jumonji C (JmjC) domain (S1 Table). For each clone, we excluded off-target edits by Sanger sequencing at the top 3 predicted off-target sites, as identified by partial complementarity to the gRNA (S3A–S3C Fig). *Kdm6a*^*-/-*^ lines do not express any KDM6A at the protein level, in contrast to *Kdm6a*^*+/+*^ controls (Figs 4B and S4A). Intriguingly, *Kdm6a*^*-/-*^ cells did not exhibit higher global levels of H3K27me3 or altered H3K4me1 compared to *Kdm6a*^*+/+*^ (Figs 4B and S4B–S4D). However, *Kdm6a*^*-/-*^ cells did have significantly higher global H3K4me3 (p = 0.030) (S4E and S4F Fig). We next differentiated the clones to chondrocytes over the course of 28 days and found that *Kdm6a*^*-/-*^ clones displayed enhanced chondrogenesis. This was visualized by increased Alcian blue staining, which labels glycosaminoglycans (GAGs) secreted by differentiated chondrocytes (Fig 4C) [29], and was subsequently quantified by increased absorbance at 605 nm (Fig 4D). Of note, we observed no difference in cell proliferation between genotypes with the MTT assay (S5 Fig); this fits with our findings in *Kdm6a*^*tm1d/+*^ and *Kdm6a*^*+/+*^ mouse growth plates (S2 Fig), which showed no difference in proliferative zone height or EdU incorporation between genotypes. Although we had observed a decrease in cross-sectional cell size in *Kdm6a*^*tm1d/+*^ growth plates (Fig 3E), it was not possible to perform such measurements *in vitro* due to technical limitations imposed by excessive matrix secretion.

To identify the transcriptional changes behind the chondrogenic phenotype in *Kdm6a*^*-/-*^ cells, we performed RNA-seq on five *Kdm6a*^*+/+*^ and five *Kdm6a*^*-/-*^ clones at day 14 of chondrocyte differentiation. We chose this time point immediately prior to the increase in Alcian blue uptake in *Kdm6a*^*-/-*^ because changes in gene expression are expected to precede phenotypic manifestation. Principal component analysis showed that the clones clustered according to genotype, with principal component 1 (PC1) accounting for 44% of inter-sample variance (Fig 4E). A total of 736 genes were differentially expressed at the 10% false-discovery rate (FDR) level (S1 Appendix). Of these, 541 genes were downregulated in *Kdm6a*^*-/-*^ chondrocytes and only 195 genes were upregulated (Fig 4F), suggesting a skew towards repression of gene expression–or possibly, loss of activation. We verified that *Kdm6a* read counts were significantly lower in *Kdm6a*^*-/-*^ chondrocytes, although transcripts could still be detected, potentially due to a time delay before the initiation of nonsense-mediated decay. *Kmt2d* was not differentially expressed in *Kdm6a*^*-/-*^ chondrocytes at the transcriptional level; we were unable to assess at the protein level due to technical limitations.

We found perturbations in chondrogenic pathways at the transcriptional level in *Kdm6a*^*-/-*^ cells. From a list of 200 genes annotated by the Mouse Genome Informatics (MGI) database as involved in cartilage development, inclusive of both positive and negative regulators (see Methods) (S2 Appendix), 164 genes were expressed in our RNA-seq dataset, and 21 had significant fold-changes (10 upregulated and 11 downregulated). These genes tended to have greater absolute fold-change in expression level than non-chondrogenic genes, suggesting that cartilage development could be collectively dysregulated in *Kdm6a*^*-/-*^ cells (S6 Fig). To quantify this effect, we showed that the p-value rank-sum of this group of genes was significantly different from the distribution of p-value rank-sums obtained from selecting groups of 164 genes at random (p < 1x10^-4^) (Fig 4G). Specific genes of interest include *Col10a1*, a marker of hypertrophic chondrocytes, which was significantly overexpressed in *Kdm6a*^*-/-*^ cells with a log_2_(fold-change) of 2.45 (Fig 4F) [30]. *Chst11*, a carbohydrate sulfotransferase responsible for the production of chondroitin sulfate, the major proteoglycan component of cartilage [31], was also significantly upregulated. Finally, *Sox6*, a transcription factor known to regulate chondrogenesis by promoting hypertrophy and organization of the growth plate [32], was also overexpressed. A number of genes annotated in the MGI cartilage development list were also downregulated. Literature investigation of several prominently downregulated genes in our data revealed that these are negative regulators of chondrogenesis (Fig 4F). For example, RSPO2 suppresses chondrocyte differentiation in a human ligament cell model [33]. While *Ogn* is present at high transcript levels in a distinct murine hypertrophic chondrocyte model, its expression drives the cells towards an osteoblastic fate, and therefore it is downregulated in our chondrogenic model system, as expected [34]. *Edn1* overexpression has been found to induce senescence in chondrocytes [35]. Altogether, these RNA-seq findings support that loss of KDM6A causes transcriptional alterations that promote excessive chondrogenesis both directly and indirectly.

We also performed Gene Ontology (GO) molecular function analysis of our RNA-seq results. Several significantly enriched GO classifiers initially looked appropriate, such as collagen binding and extracellular matrix structural constituent (S3 Appendix). Notably, most of the collagen genes within these categories actually had a negative fold-change in our data, such as *Col6a4*, *Col6a6*, *Col7a1*, *Col8a1*, and *Col15a1*. Upon further investigation in the literature, we found that these forms of collagen make up the matrix components in non-cartilaginous connective tissues, such as the basement membrane [36,37], vasculature [38], or the cornea [39]. The strong suppression of non-cartilage specific genes further suggests that *Kdm6a*^*-/-*^ cells are poised towards chondrogenesis. This stands in contrast to the aforementioned enhanced expression of *Col10a1*, a collagen type that is highly specific to hypertrophic chondrocytes in the process of endochondral ossification.

Although we wished to extend our RNA-seq findings in cell lines to our *Kdm6a*^*tm1d/+*^ mouse model, we found no significant difference between *Kdm6a*^*tm1d/+*^ and *Kdm6a*^*+/+*^ mice in protein expression of COL10A1 and EDN1 by immunofluorescence in tibial growth plates (S7A–S7D Fig). However, this may be due to the lack of sensitivity of immunofluorescence as a quantitative technique.

While chondrogenesis was a natural process to study in growth regulation, we were also interested in looking for the transcriptional signatures of other biological processes that could be perturbed by the loss of *Kdm6a*. Given our finding of decreased cell size in *Kdm6a*^*tm1d/+*^ growth plates, we performed similar p-value rank-sum analysis for positive and negative regulators of cell growth (S4 Appendix). We did not find significant dysregulation in these groups of genes (S8A and S8B Fig). Because control of cell growth and hypertrophy can vary widely between cell types, some genes annotated by MGI may not be relevant in chondrocytes, thus diluting the power of this kind of collective analysis. However, *Igf1*, a major driver of cell size, was notably downregulated in *Kdm6a*^*-/-*^ cells (S1 Appendix). Further studies are needed to clarify the contribution of the *Igf1* pathway towards the growth phenotype in KS2.

### *Kmt2d*^*-/-*^ and *Kdm6a*^*-/-*^ cell lines share a transcriptional profile indicative of excessive chondrogenesis

The similarities of precocious differentiation in both KS1 and KS2 chondrocytes prompted a direct comparison of their transcriptional profiles. We had previously generated *Kmt2d*^*-/-*^ and control *Kmt2d*^*+/+*^ clones from the same ATDC5 parental cell line by CRISPR-Cas9 gene editing. RNA-seq at Day 7 of chondrogenic differentiation revealed striking differential gene expression in *Kmt2d*^*-/-*^ lines compared to *Kmt2d*^*+/+*^ [22]. To account for the difference in timepoints and minimize batch effects, we concurrently cultured *Kmt2d*^*-/-*^ and *Kdm6a*^*-/-*^ lines and their respective wild-type control lines. Poly-adenylated RNA samples were sequenced from all samples at both Days 7 and 14 of differentiation. We first validated these results with our prior datasets. We stratified genes on the basis of significance at an FDR cutoff of 10% in our previous data, obtained at Day 7 for KS1 [22]. In our new dataset, we contrasted Day 7 *Kmt2d*^*-/-*^ samples with Day 7 *Kmt2d*^*+/+*^, and the new experiment indicates a strong enrichment of genes that were also significant in prior data (S9A Fig). Furthermore, the fold-change directionality was concordant for 864 out of 866 genes significant in both datasets (99.8%) (S9B Fig). For KS2, we performed a similar analysis stratifying genes based on prior data collected at Day 14, and again noted significant enrichment for the same genes that were previously identified (S9C Fig), as well as high concordance in the direction of fold-change (403 out of 404 genes significant in both data sets; 99.8%) (S9D Fig). As expected, at both Day 7 and Day 14, significantly fewer reads mapped to *Kmt2d* in the *Kmt2d*^*-/-*^ lines compared to *Kmt2d*^*+/+*^, while *Kdm6a* transcriptional expression was unchanged. Altogether, this confirmed that our new data aligns with prior findings where applicable, and allowed us to directly compare KS1 and KS2.

**Fig 5:**
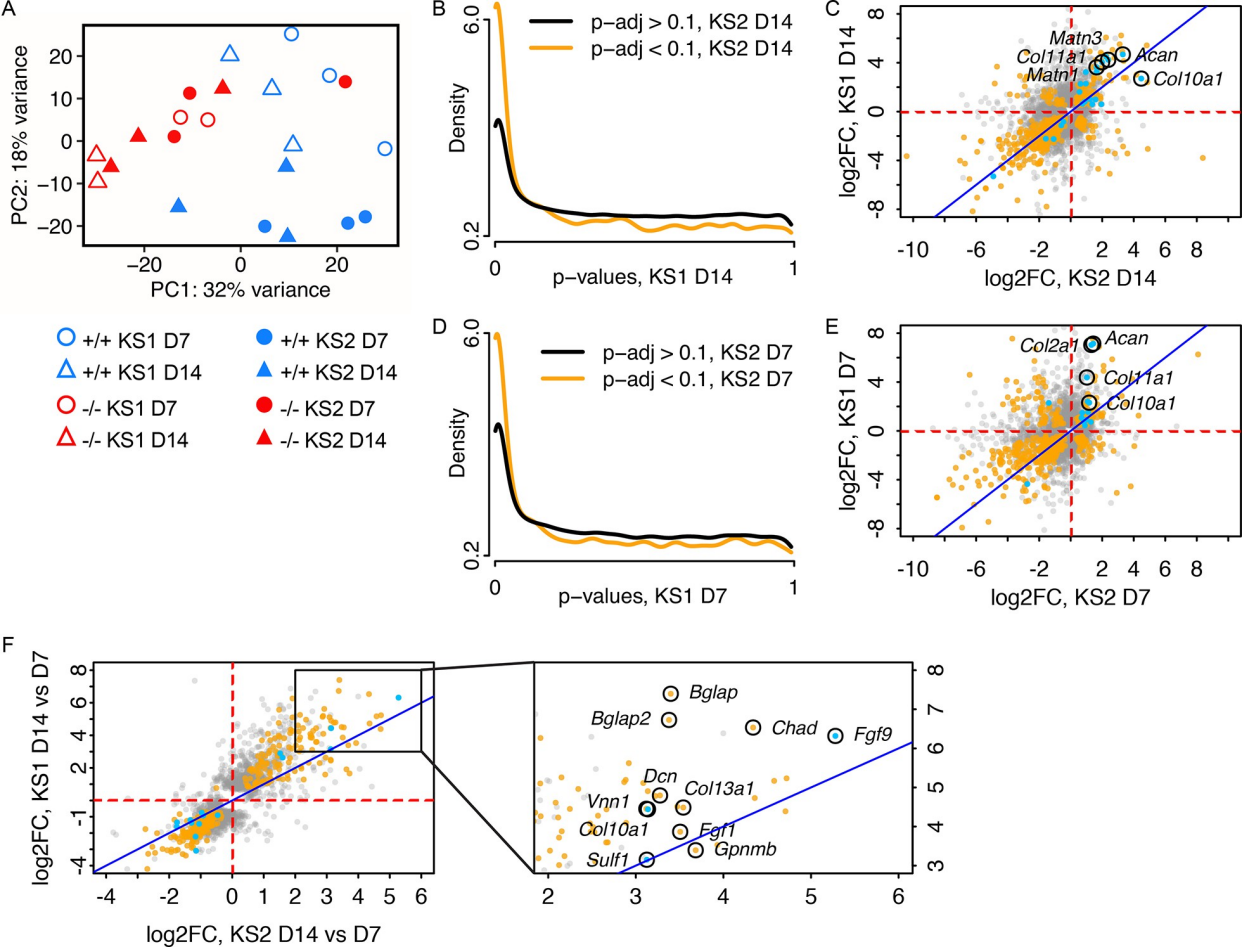
*Kmt2d*^*-/-*^ and *Kdm6a*^*-/-*^ chondrocytes bear similar transcriptomic profiles. (A) Principal component analysis of Day 7 and Day 14 RNA-seq samples from *Kmt2d*^*-/-*^ (KS1; n = 2), *Kdm6a*^*-/-*^ (KS2; n = 3), and wild-type control cell lines *Kmt2d*^*+/+*^ (KS1; n = 3) and *Kdm6a*^*+/+*^ (KS2; n = 3), respectively. Blue symbols represent wild-type; red symbols represent knockout lines. Circles represent Day 7; triangles represent Day 14. Open symbols represent KS1 (*Kmt2d*^*-/-*^ and *Kmt2d*^*+/+*^) and solid symbols represent KS2 (*Kdm6a*^*-/-*^ and *Kdm6a*^*+/+*^). (B) Conditional p-value histogram displaying p-values from the contrast of *Kmt2d*^*-/-*^ versus *Kmt2d*^*+/+*^ at Day 14 (KS1 D14), stratified by gene-wise significance at Day 14 in the contrast of *Kdm6a*^*-/-*^ versus *Kdm6a*^*+/+*^ (KS2 D14; orange line, p-adj < 0.1) or non-significance (black line, p-adj > 0.1). (C) Scatter plot of gene-wise log_2_(fold-changes) at Day 14 in the *Kdm6a*^*-/-*^ versus *Kdm6a*^*+/+*^ contrast (KS2) and the *Kmt2d*^*-/-*^ versus *Kmt2d*^*+/+*^ contrast (KS1). Orange: gene significant in both KS1 and KS2; gray: gene is significant in either KS1 or KS2, but not both; blue: gene is annotated in MGI as involved in cartilage development. Identity line (x = y) is displayed in dark blue. The (D) conditional p-value histogram is also displayed for Day 7, as is the (E) scatter plot of gene-wise log_2_(fold-changes) at Day 7. (F) Scatter plot of gene-wise log_2_(fold-changes) between Day 14 and Day 7, for KS1 and KS2. Inset panel shows a magnified area of the plot.

To investigate how factors such as genotype and differentiation stage affected sample clustering, we performed principal component analysis (PCA). This indicated separation roughly based on genotype, with principal component 1 (PC1) accounting for 32% of variance (Fig 5A). Interestingly, there was substantial variation among wild-type samples. *Kmt2d*^*+/+*^ lines that were isolated as matched controls for *Kmt2d*^*-/-*^ clones appeared to separate along principal component 2 (PC2, 18% of variance) away from *Kdm6a*^*+/+*^ controls isolated contemporaneously with *Kdm6a*^*-/-*^ lines, despite the wild-type status of both groups. We have previously confirmed by Sanger sequencing that *Kmt2d*^*+/+*^ and *Kdm6a*^*+/+*^ lines do not bear edits in the targeted loci, or in the top predicted CRISPR/Cas9 off-target sites (S3A–S3C Fig). Furthermore, PC1 accounts for nearly twice the variance as PC2 (32% versus 18%). However, in case of divergence between the two sets of wild-type samples over the course of passaging, we elected to make all further comparisons between knockout lines only with their respective matched wild-type controls, henceforth referred to as *Kmt2d*^*+/+*^ for KS1 and *Kdm6a*^*+/+*^ for KS2.

Notably, the PCA plot also showed that, for the most part, *Kmt2d*^*-/-*^ samples cluster tightly with *Kdm6a*^*-/-*^ samples regardless of timepoint (Fig 5A). To determine the overlap in gene expression patterns between KS1 and KS2 at Day 14, we compared *Kmt2d*^*-/-*^ and *Kdm6a*^*-/-*^ lines against *Kmt2d*^*+/+*^ and *Kdm6a*^*+/+*^ samples, respectively (differentially expressed genes are listed in S5 and S6 Appendices). Gene-wise p-values in KS1 were stratified by their significance in KS2 at an FDR cutoff of 10% (Fig 5B). This revealed that genes with low p-values in KS2 are likewise enriched for low p-values in KS1, suggesting a high degree of similarity between the chondrocyte transcriptomes of the two disorders. The fold-change directionality from wild-type was also extremely concordant between KS1 and KS2; of the 578 genes significant in both conditions, 139 genes were upregulated in both KS1 and KS2 and 362 of genes were downregulated, leaving only 77 genes discordant between the two conditions (Fig 5C). Mutually upregulated genes of interest include: *Acan*, which encodes aggrecan, a major proteoglycan component in cartilage; *Matn1* and *Matn3*, both encoding matrilin proteins that are part of the cartilage matrix; and several collagen genes including *Col10a1* and *Col11a1*. We also found a high degree of overlap in differentially expressed genes between KS1 and KS2 at Day 7 (Fig 5D; S7 and S8 Appendices), although fold change directionality was generally less concordant at Day 7 than at Day 14 (Fig 5E). *Acan*, *Col10a1*, *Col11a1*, and *Col2a1*, an early marker for chondrogenesis [40], were significantly upregulated in both conditions at Day 7. These results show that KS1 and KS2 have similar transcriptomic profiles, which support increased chondrocyte differentiation compared to wild-type controls.

We had performed RNA-seq at two time points, on Day 7 and Day 14 of differentiation, in order to examine the progression in gene expression in KS1 and KS2. This allowed us to assess whether the chondrocyte differentiation program differs between KS1 and KS2. We compared *Kmt2d*^*-/-*^ and *Kdm6a*^*-/-*^ samples collected at Day 14 against those collected at Day 7, and there was increased expression of a number of chondrogenic and osteogenic genes at the later time point (Fig 5F). *Fgf9*, a transcription factor that promotes chondrocyte hypertrophy [40], had 38.8-fold higher expression at Day 14 in KS1 and 79.8-fold increase in KS2, compared to Day 7. *Bglap* and *Bglap2*, which encode calcium-chelating protein hormones produced exclusively in bone tissue, were also mutually upregulated–particularly so in KS1. The overall pattern of gene expression changes from Day 7 to Day 14 indicated an increasingly chondrogenic and osteogenic environment in both *Kmt2d*^*-/-*^ and *Kdm6a*^*-/-*^ cells. Interestingly, the similarity in gene-wise fold-changes between KS1 and KS2 is greater when considering the change between time points rather than within a given time point (Fig 5C, 5E, and 5F). For instance, there is a collection of genes that are significantly upregulated in KS1 but downregulated in KS2 at Day 7, and vice versa (Fig 5E). However, with respect to the directionality of fold-change over the course of differentiation from Day 7 to Day 14, almost all significant genes are concordant between KS1 and KS2 (Fig 5F). This suggests that although individual genes may be perturbed in opposing directions at a given time point compared to wild-type, the overall trajectory of gene expression across time in KS1 and KS2 is driven by a prevailing chondrogenic differentiation program that is largely shared between the two disorders.

## Discussion

Growth abnormalities are a hallmark feature of Mendelian disorders of the epigenetic machinery (MDEMs). Here we characterized growth deficiency in a mouse model of KS2. Similar to individuals with KS2, *Kdm6a*^*tm1d/+*^ mice had reduced body length and weight. We also showed that *Kdm6a*^*tm1d/+*^ mice have shorter femurs and tibias alongside other skeletal abnormalities. Unlike in the previously published *Kmt2d*^*βgeo/+*^ model of KS1 [22], *Kdm6a*^*tm1d/+*^ mice display a decrease in growth plate height. At the cellular level, *Kdm6a*^*-/-*^ cell lines show precocious differentiation of chondrocytes, similar to *Kmt2d*^*-/-*^ lines. The increase in chondrogenesis is captured in the transcriptomic profiles of both *Kmt2d*^*-/-*^ and *Kdm6a*^*-/-*^ cells. To our knowledge, this is the first comparison of gene expression between KS1 and KS2 in a cell type related to growth deficiency.

We used cell lines that were null for *Kmt2d* or *Kdm6a* because it is generally accepted that complete loss of a protein produces a more profound effect than heterozygosity. This eased initial characterization of the phenotype and gene expression changes. However, most MDEMs–including KS1 –are dominant disorders; KS2 is X-linked. Owing to the critical functions of the epigenetic machinery, constitutional homozygous pathogenic variants have been shown to be lethal in mouse models [6,41], although conditional knockouts may be viable. Therefore, *Kmt2d*^*-/-*^ and *Kdm6a*^*-/-*^ cell lines likely capture the extreme end of the phenotypic and transcriptomic spectrum, which was displayed *in vivo* by our heterozygous mouse models that mimic human disease.

### *Kdm6a*^*tm1d/+*^ mice display a defect in longitudinal and appositional bone growth

Bone growth is closely coupled with mechanical load. Mice with lower body mass and smaller skeletal frames, as we see with the *Kdm6a*^*tm1d/+*^ genotype, thus proportionally have lower femoral cross-sectional tissue area. However, we showed that *Kdm6a*^*tm1d/+*^ mice have a significantly decreased tissue area even after normalization for shorter femur length. As tissue area can only be expanded by osteoblast activity at the periosteal surface, the lack of appropriate tissue area growth suggests that osteoblasts may be another afflicted cell type in KS2. Interestingly, femoral cortical thickness was unchanged in *Kdm6a*^*tm1d/+*^ mice and trabecular volume was increased. Decreased resorptive capability by osteoclasts at the endosteal and trabecular surfaces may also contribute towards the skeletal phenotype in KS2. Although we focus on chondrocytes here, our *in vivo* findings suggest that future studies of other cell types involved in bone growth–specifically osteoblasts and osteoclasts–would be important to expand understanding of growth deficiency in KS2.

### Recombinant human growth hormone (rhGH) is a potential therapy for KS2-associated growth deficiency

Our ultimate goal is to simultaneously treat the multiple syndromic features of KS2 by addressing the underlying molecular etiology. Specifically regarding growth, however, we believe an FDA-approved therapy, recombinant human growth hormone (rhGH), may also be efficacious in treating KS2-associated growth deficiency. Van Montfort et al. have shown that rhGH treatment improved linear growth in three individuals with KS2 and 13 individuals with KS1 [42]. Our data now provide mechanistic support for the use of rhGH in KS2 by showing that *Igf1* expression is reduced in *Kdm6a*^*-/-*^ chondrocytes and that growth plate height is reduced in *Kdm6a*^*tm1d/+*^ mice due to reduced hypertrophic chondrocyte size. This is notable because growth hormone (GH) increases linear growth both directly and indirectly through IGF1 [43,44]. In rats, GH increases *Igf1* transcript levels in the hypertrophic zone of the growth plate [45]. GH also increases growth plate height and longitudinal bone growth in mice that have undergone postnatal ablation of *Igf1r*, encoding the IGF1 receptor [46]. Therefore, rhGH would be expected to improve bone growth and height if growth deficiency in KS2 can indeed be attributed towards reduced IGF1, as our data suggest.

### Overlap between KS1 and KS2 is apparent in gene expression profiles

An extension of the Balance Hypothesis [1] supposes that impaired function of distinct components of the epigenetic machinery may yet converge upon the same phenotype by achieving a shared chromatin state and gene expression. We found that despite the distinct genetic etiologies of KS1 and KS2, downstream gene expression patterns appear to be shared, which has also been noted by others. Luperchio et al. used RNA-seq on B lymphocytes isolated from mouse models of KS1 and KS2 and found a high degree of overlap in differential gene expression [18]. Several groups have developed a genome-wide DNA methylation signature in peripheral blood that can differentiate individuals with KS1 from healthy controls [47–49]. Interestingly, this signature also identifies KS2 samples due to the high degree of overlap, and has even aided in diagnosis [47,50,51]. As gene expression is dependent upon DNA methylation status, this suggests that there is a shared ‘Kabuki transcriptome’ in peripheral blood. Relative to wild-type controls, we showed striking concordance in gene expression between *Kmt2d*^*-/-*^ and *Kdm6a*^*-/-*^ cells at multiple stages of chondrocyte differentiation. The transcriptional signatures of these genotypes were both suggestive of precocious chondrogenesis. This also matched our cellular phenotype of increased Alcian blue staining in *Kdm6a*^*-/-*^ lines, described here, and our previously published findings in *Kmt2d*^*-/-*^ lines [22].

### KS1 and KS2 may lead to growth deficiency through distinct pathways

Several pieces of data indicate that the mechanism of growth deficiency in KS1 may still differ from KS2 in subtle ways. Our prior work identified *Shox2* as a direct target of KMT2D [22]. *Shox2* is expressed in mouse growth plates, and conditional loss of *Shox2* in chondrocytes causes shortened limbs [52,53]. *Kmt2d*^*-/-*^ cells exhibited depletion of H3K4me3 at the *Shox2* promoter relative to *Kmt2d*^*+/+*^ lines, concurrent with a 4-fold downregulation of *Shox2* expression. The decrease in SHOX2 led to a disinhibition of *Sox9* expression in *Kmt2d*^*-/-*^ cells. As SOX9 is a pro-chondrogenic transcription factor [54,55], we proposed that this pathway is implicated in the precocious differentiation of chondrocytes, which we have shown to be a common feature of KS1 and KS2. Yet in *Kdm6a*^*-/-*^ cells, neither *Shox2* nor *Sox9* is differentially expressed relative to *Kdm6a*^*+/+*^ controls, at either Day 7 or Day 14 of chondrogenic differentiation. This suggests that the *Shox2-Sox9* pathway is not a driving factor in precocious chondrogenesis in KS2. Notably, even in *Kmt2d*^*-/-*^ cells, repletion of *Shox2* by lentiviral overexpression was insufficient to restore Alcian blue staining to wild-type levels [22], indicating that additional pathways–possibly shared between KS1 and KS2 –contribute towards this phenotype.

A caveat to these conclusions is that our *Kmt2d*^*+/+*^ and *Kdm6a*^*+/+*^ control lines, both previously confirmed to be wild-type, appear to differ at baseline. Whereas this may be due to passaging effects, as the clones were derived on separate instances, or to the slightly different CRISPR methods used to generate the lines, it remains possible that the aforementioned gene expression differences between KS1 and KS2 could be an artifact of our imperfect cell models. However, detailed examination of skeletal profiles in KS1 and KS2 mouse models also reveals differences despite the preserved global picture of growth deficiency. Compared to wild-type littermates, *Kmt2d*^*βgeo/+*^ and *Kdm6a*^*tm1d/+*^ mice have significantly lower body weight and body length, and shorter femurs and tibias [22]. Unlike *Kdm6a*^*tm1d/+*^ mice, however, *Kmt2d*^*βgeo/+*^ mice did not have any significant difference in tissue area, and trabeculae were thinner compared to wild-type. In the tibial growth plate, *Kdm6a*^*tm1d/+*^ mice had reduced hypertrophic chondrocyte size, leading to a significant decrease in hypertrophic zone and overall growth plate height. This was the opposite of growth plate findings in *Kmt2d*^*βgeo/+*^ mice. These data definitively point to chondrocytes as a critical cell type in the pathogenesis of growth deficiency for both KS1 and KS2. However, it remains a question how to reconcile opposing growth plate data with the ultimate shared phenotype of shortened femurs and tibias.

### Features of KS1 and KS2 mice recur in mouse models of other genetic diseases

We are not aware of other mouse models with growth plate findings identical to KS1 or KS2 mice that definitively suggest a common mechanism. However, there are reported mouse models that resemble certain aspects of each. In fact, both shortened and expanded growth plates have occurred in association with decreased long bone length. A thanatophoric dysplasia type 1 mouse model containing the hypermorphic *Fgfr3*^*Y367C/+*^ variant showed shortened long bones and reduced hypertrophic zone height due to reduced cell size [56], similar to our *Kdm6a*^*tm1d/+*^ mice. However, the growth plates were also severely disorganized, unlike in *Kdm6a*^*tm1d/+*^ mice. Of note, the genes encoding two FGFR3 ligands, *Fgf1* and *Fgf9*, are upregulated in KS1 and KS2 chondrocytes, supporting a role in activation of this pathway in both disorders. Similarly, we are not aware of a disorder that completely mimics our findings in KS1 mice. *Dmp1*^*-/-*^ mice partially resemble KS1, with shortened long bones and expanded growth plates involving both the proliferative and hypertrophic zones [57]. Distinct from our previous findings in KS1 [22], *Dmp1*^*-/-*^ mice also show reduced apoptosis of hypertrophic chondrocytes [57].

### Chondrocyte-to-osteoblast transdifferentiation remains to be explored in KS1 and KS2

Altered cell differentiation is a common disease process in this class of disorders, as described in other MDEMs [22,58,59]. Cell type-specific gene expression is generally controlled through epigenetics, and prior studies indicate that disruptions to the epigenetic machinery impact cell fate and commitment [60]. The classical model of endochondral ossification describes apoptosis of hypertrophic chondrocytes and subsequent invasion of the residual cartilage template by osteoblasts. Accelerated chondrocyte differentiation, as appears to occur in KS1 and KS2, could therefore impact downstream processes of bone formation. Not only might an altered epigenome hasten or delay differentiation, it is also conceivable that the barriers between cell types may become increasingly fluid. Chondrocytes and osteoblasts were originally believed to be mutually exclusive, committed cell fates resulting from differentiation of mesenchymal stem cells [61]. However, lineage tracing experiments using transgenic mice have now led to several hypotheses for chondrocyte-to-osteoblast transdifferentiation [62]. Whereas some groups propose transdifferentiation at the immature chondrocyte stage [63], others suggest dedifferentiation of hypertrophic chondrocytes followed by redifferentiation [64–66], and yet additional data may support direct transdifferentiation from hypertrophic chondrocytes into mature osteoblasts [67–69]. A disruption in this complex interplay of cell fates could plausibly contribute to growth plate differences in KS1 and KS2. We noted an extreme upregulation of *Bglap* and *Bglap2* between Day 7 and Day 14 of chondrocyte differentiation in *Kmt2d*^*-/-*^ cell lines in particular. Alternatively named *Osteocalcin*, these genes are regarded to be osteoblast-specific [70], suggesting that transdifferentiation may be occurring. *Kdm6a*^*-/-*^ and wild-type cell lines also increased expression of these genes over the course of differentiation, although to a lesser degree. ATDC5 cells are an established *in vitro* model system for chondrogenesis [26–28]. However, it would be ideal to further investigate *in vivo* whether KS1 and/or KS2 change the potential for chondrocyte-to-osteoblast transdifferentiation, as an imbalance in these cell types would impact the bone environment and appositional bone growth.

### KMT2D and KDM6A co-exist in the COMPASS complex

The Balance Hypothesis is an appealing explanation for the clinical resemblance of KS1 and KS2, but it is important to consider alternative hypotheses as well. In support for the former, Björnsson et al. have shown that *Kmt2d*^*βgeo/+*^ mice have genome-wide depletion of H3K4me3, which is the expected direct consequence of reduced KMT2D activity [71]. This was reversible upon treatment with a histone deacetylase inhibitor (HDACi), AR-42, which also ameliorated neurologic findings of KS1 in the mice. These data, in combination with the Kabuki syndrome DNA methylation signatures [47–49], point towards an epigenetic etiology for these disorders. Luperchio et al. also found that *Kmt2d*^*βgeo/+*^ and *Kdm6a*^*tm1d/+*^ B lymphocytes both tend towards increased promoter accessibility [18]. On the one hand, this is supportive of KS1 and KS2 having a shared chromatin state that contributes to overlapping gene expression, which we saw in our RNA-seq data comparing *Kmt2d*^*-/-*^ and *Kdm6a*^*-/-*^ cells. Yet, loss of either a writer of H3K4me3 or an eraser of H3K27me3 would be expected to have the opposite effect on chromatin accessibility. Two potential possibilities (which are not mutually exclusive) include: 1. An indirect effect that is downstream of KMT2D or KDM6A catalytic activity drives the determination of chromatin state, or 2. The role of KMT2D and/or KDM6A in the pathogenesis of Kabuki syndrome stems from a non-catalytic function in one or both of these proteins.

Our western blot results show that global H3K27me3 levels are unaltered even in *Kdm6a*^*-/-*^ cells with no residual KDM6A protein. This is not entirely unsurprising, as the KDM6 family also includes KDM6B, which shares 70% protein sequence identity with KDM6A and also has H3K27 demethylase activity [12]. However, it raises the possibility that the predominant role of KDM6A in KS2 may be non-catalytic. KMT2D and KDM6A coexist in the COMPASS complex, which broadly serves as an antagonist to gene repression by Polycomb group proteins throughout development [72]. The reduced dosage of either KMT2D or KDM6A could disrupt stoichiometry or destabilize the COMPASS complex–and perhaps the shared features of KS1 and KS2 are rooted in the destabilization of COMPASS rather than the individual enzymatic functions of KMT2D and KDM6A. KDM6A is destabilized in *Kmt2d*^*-/-*^ mouse embryonic stem cells, suggesting interdependency of KDM6A upon KMT2D expression [73]. Due to technical limitations, we were unable to verify the converse at the protein level, although we noted that *Kmt2d* expression is unaltered at the transcriptional level in KS2 chondrocytes. Interestingly, global H3K4me3 is increased in *Kdm6a*^*-/-*^ cells. Given that KMT2D is a writer of H3K4me3, this result initially appears to contradict the hypothesis of interdependency between KDM6A and KMT2D. However, KMT2D is not the sole methyltransferase to target H3K4 [74], and it remains possible that gene expression changes resulting from loss of KDM6A and/or KMT2D could impact another component of the epigenetic machinery, thus indirectly upsetting the balance of global H3K4me3 levels.

Domain-swapping experiments have shown that KDM6A has a non-catalytic capacity to recruit the histone acetyltransferase p300 to co-localize with COMPASS at enhancer elements [75]. Likewise, catalytic-dead KDM6A is sufficient to rescue differentiation in *Kdm6a*-knockout mouse embryonic stem cells [24,76], establishing that KDM6A plays important non-enzymatic roles. It would be interesting to consider the formation and genomic localization of the COMPASS complex in the absence or reduced dosage of KMT2D, as well as KDM6A. Should the catalytic role of KMT2D also be implicated in the pathogenesis of KS2, it may unlock the possibility of treatment with pharmacological and/or dietary therapies, which have already been shown to improve neurological outcomes in *Kmt2d*^*βgeo/+*^ mice [71,77,78].

### Summary

In this work, we aimed to further understand the mechanisms involved in growth deficiency for two related MDEMs, KS1 and KS2. We showed that while skeletal and growth plate findings differ in certain aspects between mouse models of KS1 and KS2, chondrocytes bearing *Kmt2d* or *Kdm6a* null mutations exhibit a shared phenotype of precocious differentiation. Importantly, the similarity in transcriptional profiles raises the tantalizing possibility of developing a treatment to target both disorders.

## Methods

### Ethics statement

The Johns Hopkins Institutional Animal Care and Use Committee approved all animal studies in this project. Procedures were performed following standards described in the NIH *Guide for the Care and Use of Laboratory Animals* (National Academies Press, 2011).

### Animals

The *Kdm6a*^*tm1a(EUCOMM)Wtsi*^ allele (*tm1a*) was designed by the International Knockout Mouse Consortium (IKMC) and consisted of a *FRT*-flanked LacZ-neomycin resistance cassette (*βgeo*), upstream of the *lox*P-flanked *Kdm6a* exon 3. *Kdm6a*^*tm1a/+*^ females were obtained by the Björnsson lab from the European Mutant Mouse Archive (EMMA) and crossed with B6.Cg-Tg(ACTFLPe)9205Dym/J males (Strain No. 005703, The Jackson Laboratory), which express *FLP1* driven by the *Actb* promoter. This excised the *βgeo* cassette and generated the *tm1c* allele. *Kdm6a*^*tm1c/tm1c*^ females were next crossed with B6.C-Tg(CMV-cre)1Cgn/J males (Strain No. 006054, The Jackson Laboratory), which carry a human CMV-driven *cre* transgene integrated on chromosome X. *Cre*-mediated excision of *Kdm6a* exon 3 resulted in the *tm1d* allele. *Kdm6a*^*tm1d/+*^ females were backcrossed to C57BL/6J males (Strain No. 000664, The Jackson Laboratory) to remove *FLP1* and *cre* transgenes and to maintain the strain subsequently. Due to the perinatal lethality of *Kdm6a*^*tm1d/y*^ males, the *tm1d* allele can only be maintained in the female line. Therefore, all experimental animals were bred by crossing *Kdm6a*^*tm1d/+*^ females with wild-type C57BL/6J males. Genotyping was performed by PCR. All mice were in the care of Johns Hopkins Research Animal Resources. Up to 5 mice were housed in each barrier cage with *ad libitum* access to autoclaved feed (Envigo Teklad 2018SX) and reverse osmosis-filtered, hyperchlorinated water. A cotton square nestlet (Envigo Teklad 6060/6105) and autoclaved corncob bedding (Envigo Teklad 7092/7097) were provided for each cage. Cages were changed every 2 weeks under aseptic conditions. Cage racks were ventilated with HEPA-filtered and humidified air. Animal rooms were maintained on standard light/dark cycles at regulated temperatures. Mice were euthanized by halothane inhalation (Sigma-Aldrich B4388), following the AVMA Guidelines for the Euthanasia of Animals, 2020 edition (https://olaw.nih.gov/policies-laws/avma-guidelines-2020.htm).

### High-resolution micro-computed tomography

Mice were euthanized at 8 weeks of age. Femurs and tibias were dissected and fixed with 4% paraformaldehyde in 1x PBS for 24–48 hours at 4°C, then transferred to 70% ethanol. Digital calipers were used to measure bone length. Imaging was performed following the guidelines of the American Society for Bone and Mineral Research [79]. A Bruker Skyscan 1172 desktop microcomputed tomography system was used to scan bones at 65 keV and 152 μA with a 0.5 mm aluminum filter at an isotropic voxel size of 10 μm. Image reconstruction was performed with nRecon (Bruker), and analysis was done with CTAn software (Bruker). A 500 μm region of interest (ROI) centered around the femoral mid-diaphysis was assessed for cortical bone measurements. Trabecular measurements were collected 500 μm proximal to the distal femoral growth plate in a 2 mm ROI. All micro-CT imaging and measurements were carried out under blinded conditions.

### Growth plate measurements

Femurs and tibias dissected from 8-week-old mice were fixed with 4% paraformaldehyde in 1x PBS for 24–48 hours at 4°C, then decalcified by continual agitation in a 14% EDTA solution, pH 7.4 (Bio-Rad 1610729) at 4°C. Decalcifying solution was refreshed every 24–48 hours until bones were pliable. Bones were processed by the Johns Hopkins Hospital Reference Histology Laboratory and embedded in paraffin blocks. Hematoxylin and eosin staining (H&E) was performed on mounted 5 μm sections of the proximal tibial growth plates. Images were taken with a Nikon 80i microscope under brightfield illumination at 10, 20, and 40x magnification and analyzed using NIS elements software to obtain height measurements of the proliferative zone (PZ), hypertrophic zone (HZ), and growth plate (GP). Measurements were averaged over 3 sites per section, for 4 sections per sample. Cell counts were obtained in the proliferative and hypertrophic zones and averaged for each mouse. Cell size was assessed for hypertrophic chondrocytes using ImageJ and averaged over 15 cells per section, 4 sections per mouse (60 cells total). All imaging and growth plate measurements were performed blinded to the genotype.

### EdU incorporation assay

*Kdm6a*^*tm1d/+*^ and *Kdm6a*^*+/+*^ mice were injected with 150 mg/kg of EdU at the age of 8 weeks. Twelve to sixteen hours post-injection, the mice were euthanized. The long bones were dissected and fixed with 4% paraformaldehyde in 1X PBS for 36 hours, before demineralization with 14% EDTA in 1X PBS as described above. Bones were embedded in paraffin blocks and 5 μm sections of proximal tibial growth plates were adhered to glass slides. Sections were deparaffinized in xylene and rehydrated in decreasing concentrations of ethanol. EdU incorporation was detected using the Click-iT EdU Cell Proliferation Kit (ThermoFisher Scientific C10337) and nuclei were counterstained with Hoechst dye.

### Growth plate immunofluorescence

Paraffin-embedded longitudinal proximal tibial growth plate sections were deparaffinized and rehydrated. Antigen retrieval was performed with 0.05% citraconic anhydride at pH 7.4 [80]. Slides were then rinsed and permeabilized, blocked with 5% goat serum and then incubated overnight in primary antibody. Following washes, slides were then incubated in secondary antibody. Excess antibody was washed off before slides were stained with DAPI. Quantification was performed by taking the total fluorescence intensity per DAPI-stained cell count in the growth plate in ImageJ. Background subtraction was performed using the rolling ball algorithm with radius size 150 pixels and cell counts were performed in ImageJ. All antibodies are listed in S2 Table. All images and measurements were performed blinded.

### ATDC5 cell culture

ATDC5 cells were sourced from the European Collection of Authenticated Cell Cultures (Sigma-Aldrich 99072806). Cells were propagated at 37°C, 5% CO_2_, in ATDC5 Complete Media. This consisted of DMEM/F-12 (ThermoFisher Scientific 11320033) supplemented with 5% heat-inactivated fetal bovine serum (ThermoFisher Scientific 16140071), 2 mM L-glutamine (Corning 25-005-CI), 100 U/mL penicillin and 100 μg/mL streptomycin (ThermoFisher Scientific 15140122). Media was changed every 48–72 hours.

### Chondrogenic differentiation

24 hours post-seeding, media was exchanged for ATDC5 Complete Media supplemented with 0.05 mg/mL L-ascorbic acid (Sigma-Aldrich A4403), 10 mM β-glycerophosphoric acid (ThermoFisher Scientific 410991000), and 1x Insulin-Transferrin-Selenium (ITS-G) (ThermoFisher Scientific 41400045). Cells were cultured for up to 28 days at 37°C, 5% CO_2_, with supplemented media changes every 48–72 hours.

### Generation of *Kdm6a*^*-/-*^ cell lines

The crRNA was designed to target exon 6 of *Mus musculus Kdm6a* with the following guide sequence: 5’-TTCCTTGGCTCGACAAAAGC-3’. Alt-R CRISPR-Cas9 tracrRNA and Alt-R custom Cas9 crRNA (Integrated DNA Technologies) were mixed at an equimolar ratio in Nuclease-Free Duplex buffer (Integrated DNA Technologies 11-01-03-01) and allowed to anneal into duplexes. Alt-R S.p. Cas9 Nuclease V3 (Integrated DNA Technologies 1081058) was introduced for duplex loading. The resulting ribonucleoprotein (RNP) complex was incubated with CRISPRMAX (ThermoFisher Scientific CMAX00001) and Opti-MEM (ThermoFisher Scientific 31985062) for formation of transfection complexes. Meanwhile, ATDC5 cells were rinsed with 1x PBS, dissociated with 0.25% trypsin, 2.21 mM EDTA (Corning 25-053-CL), and collected for counting. Cells were seeded at 2x10^5^ cells per well in 6-well plates concurrently with introduction of the RNP transfection complexes. After 48 hrs at 37°C, 5% CO_2_, cells were washed and trypsinized. A sample was lysed with QuickExtract DNA Extraction Solution (Biosearch Technologies QE09050) to assess editing efficiency with the Alt-R Genome Editing Detection Kit (Integrated DNA Technologies 1075932). Remaining cells were seeded either in 96-well plates at 1 cell per 50 μL (limiting dilution cloning), or in 10cm dishes at low densities to obtain single colonies. After 10 days of growth, cells were washed with 1x PBS and underwent mild dissociation in warm trypsin for 1 minute. Single colonies were visually identified, isolated, and expanded to establish clonal lines. Successfully edited lines and unedited control lines were identified using the Alt-R Genome Editing Detection Kit and verified by Sanger sequencing. Forward primer: 5’-AAGATAGAGTGCAGTGGGTTG-3’. Reverse primer: 5’-CAGAAGTCCAAATGCCTTGTAAAT-3’.

### Generation of *Kmt2d*^*-/-*^ cell lines

Please refer to Fahrner et al., 2019 [22].

### Western blot

Cells were washed with cold 1x PBS twice and lysed with RIPA buffer containing protease inhibitor (ThermoFisher Scientific A32953) to isolate total protein. Histones were extracted using a kit following manufacturer’s protocols (Abcam ab113476). The Pierce BCA Protein Assay kit (ThermoFisher Scientific 23225) was used to measure protein concentrations, and 10 μg of each sample was loaded onto a NuPAGE 4 to 12%, Bis-Tris gel (ThermoFisher Scientific NP0336BOX). Following transfer of proteins, PVDF membranes were blocked with 0.5x Intercept (PBS) Blocking Buffer (LI-COR Biosciences 927–70001) for 1 hour at room temperature. Membranes were stained with primary antibodies overnight at 4°C and repeatedly washed with 1x PBS with 0.1% Tween-20 (PBS-T) the following day. Afterwards, membranes were incubated in secondary antibody for one hour at room temperature. Excess antibody was removed with PBS-T washes. Imaging was performed with the LI-COR Odyssey. All antibodies are listed in S2 Table. Image Studio version 5.2 was used to quantify blots by densitometry.

### Alcian blue staining

Cells were seeded at a density of 1x10^5^ cells per well in 6-well plates (Corning 353046). Day 7–28 wells underwent chondrogenic differentiation for the specified number of days; Day 0 wells were collected 24 hours post-seeding. Cells were rinsed with 1x PBS and fixed for 5 minutes in 10% neutral buffered formalin (Epredia 9990244). After 3 washes in 1x PBS to remove residual fixative, cells were stained with 1% Alcian blue solution in 3% acetic acid, pH 2.5 (Sigma-Aldrich B8438) for 1 hour in the dark. Unbound dye was removed with 3 washes in 1x PBS. Whole-well images were captured over a transilluminator. An EVOS FL Auto Imaging System (ThermoFisher Scientific) was used to obtain images at 4X, 10X, and 20X magnification. For quantification of the Alcian blue stain, well contents were lysed in a 10% sodium dodecyl sulfate solution (Sigma-Aldrich L3771) for 20 minutes at minimum. Absorbance was measured at 605 nm with a BioTek Synergy 2 Multi-Mode Microplate Reader.

### MTT assay

Cells were seeded at a density of 1x10^3^ cells per well in 96-well plates (Corning 353072). Day 7–28 wells underwent chondrogenic differentiation for the specified number of days; Day 0 wells were collected 24 hours post-seeding. 50 μL of ATDC5 Complete Media and 10 μL of CellTiter 96 AQueous One Solution (Promega G3580) were added to each well. Plates were incubated at 37°C, 5% CO_2_ for 1.5 hours to allow colorimetric conversion to the formazan product. Absorbance was measured at 490 nm with a BioTek Synergy 2 Multi-Mode Microplate Reader.

### RNA isolation

Cells were seeded at a density of 1x10^5^ cells per well in 6-well plates (Corning 353046) and underwent chondrogenic differentiation for the specified number of days. Samples were collected with TRIzol reagent (ThermoFisher Scientific 15596026) and chloroform. Following phase separation, the aqueous phase containing RNA was put through the RNA Clean & Concentrator-5 kit (Zymo Research R1013) with DNase I digestion to remove genomic DNA contamination.

### RNA-sequencing library preparation

RNA quantity was measured by Qubit RNA Broad Range Assay (ThermoFisher Scientific Q10210). RNA quality was assessed on an Agilent Fragment Analyzer by the Johns Hopkins Single Cell & Transcriptomics Core (JH SCTC) Facility. 1 μg of total RNA per sample was used as input for the NEBNext Ultra II RNA Library Prep kit with Sample Purification Beads (E7775S), in combination with the NEBNext Poly(A) mRNA Magnetic Isolation Module (New England BioLabs E7490L). Libraries were indexed with NEBNext Multiplex Oligos for Illumina (Dual Index Primers Set 1) (New England BioLabs E7600S). Completed libraries underwent quality assessment on the Agilent Fragment Analyzer run by JH SCTC, and were quantified with the NEBNext Library Quant Kit for Illumina (New England BioLabs E7630L). Libraries were pooled to a final concentration of 4 nM and submitted to the Johns Hopkins Genomics Research Core Facility for high-throughput sequencing on the Illumina NovaSeq 6000 platform, using SP flow cells to generate 100-bp paired-end reads.

### Analysis of RNA-sequencing data

The GRCm38 transcriptome was indexed using Salmon 1.9.0 [81], using the GRCm38 genome as the decoy sequence (Mus_musculus.GRCm38.cdna.all.fa.gz and Mus_musculus.GRCm38.dna.primary_assembly.fa.gz, from http://nov2020.archive.ensembl.org/Mus_musculus/Info/Index, release 102). FASTQ files of demultiplexed paired-end reads were mapped using Salmon 1.9.0, with selective alignment and GC bias correction. Transcript quantification files were loaded into R 4.1.2, running Bioconductor 3.14, with tximeta 1.12.4 [82], and transcript-level counts were summarized by gene. Gene-level counts were aggregated for any technical replicates and filtered for non- or low-expressed genes (median count over samples < 10). Surrogate variable (SV) analysis was performed using sva 3.42.0 [83] for the dual experiment between *Kmt2d*^*-/-*^ and *Kdm6a*^*-/-*^ cell lines, identifying up to 2 SVs in certain contrasts. Differential expression analysis was performed using DESeq2 1.34.0 [84] with a false-discovery rate threshold < 0.1. biomaRt 2.50.3 was used to annotate genes according to Ensembl *Mus musculus* version 102 [85]. Differentially expressed genes are listed in S1 and S5–S8 Appendices. For the comparison to prior Kabuki syndrome 1 data, raw reads were downloaded from the Gene Expression Omnibus (GEO), accession number GSE129365, and processed identically. The Mouse Genome Informatics (MGI) database was accessed on July 11, 2023, to download a list of genes falling under the Gene Ontology (GO) category of ‘cartilage development’. Annotations for negative regulators of this process were removed, leaving 200 genes (complete list in S2 Appendix). MGI was again accessed on March 12, 2024, to download lists of genes within the GO categories of ‘positive regulation of cell size’ and ‘negative regulation of cell size’ (S4 Appendix). A two-sample Wilcoxon test statistic was calculated for the p-values of genes included in these lists and compared against the distribution of test statistics obtained from performing this test on random groups of genes for 10,000 iterations.

### Gene Ontology analysis

PANTHER overrepresentation test (release 2024-02-26) was performed with the GO Ontology molecular function database (release 2024-01-17) using the list of differentially expressed genes in *Kdm6a*^*-/-*^ cells compared to *Kdm6a*^*+/+*^ at Day 14. The reference gene list comprised all expressed genes in the dataset (median count over samples > 10).

### Statistics

With exception of the RNA-seq data analysis (which is described in Methods, ‘Analysis of RNA-sequencing data’), two-tailed, unpaired Student’s t-tests were used with a cutoff of p < 0.05.

## Supporting information

S1 Fig Tissue area of *Kdm6a*^*tm1d/+*^ and *Kdm6a*^*+/+*^ littermates, normalized by femur length. (PDF)

S2 Fig Cell proliferation rate does not differ between growth plates from *Kdm6a*^*tm1d/+*^ and *Kdm6a*^*+/+*^ mice. (PDF)

S3 Fig Absence of off-target editing in *Kdm6a*^*+/+*^ and *Kdm6a*^*-/-*^ cell lines at top predicted exonic sites. (PDF)

S4 Fig *Kdm6a*^*-/-*^ cells do not express KDM6A and have increased global H3K4me3. (PDF)

S5 Fig *Kdm6a*^*-/-*^ cells do not differ in proliferation rate from *Kdm6a*^*+/+*^. (PDF)

S6 Fig Density distribution of expression fold-changes comparing *Kdm6a*^*-/-*^ to *Kdm6a*^*+/+*^ chondrocytes. (PDF)

S7 Fig Growth plates from *Kdm6a*^*tm1d/+*^ and *Kdm6a*^*+/+*^ mice do not differ in protein expression of EDN1 and COL10A1. (PDF)

S8 Fig *Kdm6a*^*-/-*^ cells do not exhibit collective dysregulation of cell size pathways. (PDF)

S9 Fig Validation of KS1 and KS2 RNA-seq data by comparison to prior datasets. (PDF)

S1 Table ATDC5 cell line genotypes. *Kdm6a*^*-/-*^ frameshift variants generated using CRISPR/Cas9 gene editing.
(PDF)

S2 Table Reagents. Detailed list of reagents used in the manuscript.
(PDF)

S1 Appendix KS2 differentially expressed genes, Day 14. The 736 differentially expressed genes at the 10% false-discovery rate (FDR) level between *Kdm6a*^*-/-*^ and *Kdm6a*^*+/+*^ after 14 days of differentiation towards chondrocytes.
(XLSX)

S2 Appendix MGI Cartilage Development genes. List of 200 genes involved in cartilage development from the Mouse Genome Informatics (MGI) database.
(XLSX)

S3 Appendix Gene Ontology (GO) molecular pathway analysis for KS2 differentially expressed genes, Day 14. Complete list of significantly enriched GO terms among the differentially expressed genes between *Kdm6a*^*-/-*^ and *Kdm6a*^*+/+*^ cells at Day 14 of chondrocyte differentiation, using all expressed genes in the dataset as the reference file.
(XLSX)

S4 Appendix MGI positive and negative regulators of cell size. List of 89 genes annotated as positive regulators and 125 genes annotated as negative regulators, from the Mouse Genome Informatics (MGI) database.
(XLSX)

S5 Appendix KS1 and KS2 combined RNA-seq experiment differentially expressed genes: *Kmt2d*^*-/-*^ vs *Kmt2d*^*+/+*^, Day 14. List of differentially expressed genes in *Kmt2d*^*-/-*^ vs *Kmt2d*^*+/+*^ chondrocytes after 14 days of differentiation.
(XLSX)

S6 Appendix KS1 and KS2 combined RNA-seq experiment differentially expressed genes: *Kdm6a*^*-/-*^ vs *Kdm6a*^*+/+*^, Day 14. List of differentially expressed genes in *Kdm6a*^*-/-*^ vs *Kdm6a*^*+/+*^ chondrocytes after 14 days of differentiation.
(XLSX)

S7 Appendix KS1 and KS2 combined RNA-seq experiment differentially expressed genes: *Kmt2d*^*-/-*^ vs *Kmt2d*^*+/+*^, Day 7. List of differentially expressed genes in *Kmt2d*^*-/-*^ vs *Kmt2d*^*+/+*^ chondrocytes after 7 days of differentiation.
(XLSX)

S8 Appendix KS1 and KS2 combined RNA-seq experiment differentially expressed genes: *Kdm6a*^*-/-*^ vs *Kdm6a*^*+/+*^, Day 7. List of differentially expressed genes in *Kdm6a*^*-/-*^ vs *Kdm6a*^*+/+*^ chondrocytes after 7 days of differentiation.
(XLSX)

S1 Data Raw data values for all graphs in manuscript and supplemental information. (XLSX)

## References

[1] Fahrner JA, Bjornsson HT. Mendelian disorders of the epigenetic machinery: Tipping the balance of chromatin States. Annu Rev Genomics Hum Genet. 2014;15:269–93. doi: 10.1146/annurev-genom-090613-094245 25184531 PMC4406255

[2] Adam MP, Banka S, Bjornsson HT, Bodamer O, Chudley AE, Harris J, et al. Kabuki syndrome: International consensus diagnostic criteria. J Med Genet. 2019;56(2):89–95. doi: 10.1136/jmedgenet-2018-105625 30514738

[3] Bögershausen N, Gatinois V, Riehmer V, Kayserili H, Becker J, Thoenes M, et al. Mutation Update for Kabuki Syndrome Genes KMT2D and KDM6A and Further Delineation of X-Linked Kabuki Syndrome Subtype 2. Hum Mutat. 2016;37(9):847–64. doi: 10.1002/humu.23026 27302555

[4] Ng SB, Bigham AW, Buckingham KJ, Hannibal MC, McMillin MJ, Gildersleeve HI, et al. Exome sequencing identifies MLL2 mutations as a cause of Kabuki syndrome. Nat Genet. 2010;42(9):790–3. doi: 10.1038/ng.646 20711175 PMC2930028

[5] Dhar SS, Lee SH, Kan PY, Voigt P, Ma L, Shi X, et al. Trans-tail regulation of MLL4-catalyzed H3K4 methylation by H4R3 symmetric dimethylation is mediated by a tandem PHD of MLL4. Genes Dev. 2012;26(24):2749–62. doi: 10.1101/gad.203356.112 23249737 PMC3533079

[6] Lee JE, Wang C, Xu S, Cho YW, Wang L, Feng X, et al. H3K4 mono- And di-methyltransferase MLL4 is required for enhancer activation during cell differentiation. Elife. 2013;2013(2):1–25. doi: 10.7554/eLife.01503 24368734 PMC3869375

[7] Rada-Iglesias A. Is H3K4me1 at enhancers correlative or causative? Nat Genet. 2018;50(1):4–5. doi: 10.1038/s41588-017-0018-3 29273804

[8] Murakami H, Tsurusaki Y, Enomoto K, Kuroda Y, Yokoi T, Furuya N, et al. Update of the genotype and phenotype of KMT2D and KDM6A by genetic screening of 100 patients with clinically suspected Kabuki syndrome. Am J Med Genet Part A. 2020;182(10):2333–44. doi: 10.1002/ajmg.a.61793 32803813

[9] Lederer D, Grisart B, Digilio MC, Benoit V, Crespin M, Ghariani SC, et al. Deletion of KDM6A, a histone demethylase interacting with MLL2, in three patients with kabuki syndrome. Am J Hum Genet. 2012;90(1):119–24. doi: 10.1016/j.ajhg.2011.11.021 22197486 PMC3257878

[10] Miyake N, Koshimizu E, Okamoto N, Mizuno S, Ogata T, Nagai T, et al. MLL2 and KDM6A mutations in patients with Kabuki syndrome. Am J Med Genet Part A. 2013;161(9):2234–43. doi: 10.1002/ajmg.a.36072 23913813

[11] Agger K, Cloos PAC, Christensen J, Pasini D, Rose S, Rappsilber J, et al. UTX and JMJD3 are histone H3K27 demethylases involved in HOX gene regulation and development. Nature. 2007;449(7163):731–4. doi: 10.1038/nature06145 17713478

[12] Hong SH, Cho YW, Yu LR, Yu H, Veenstra TD, Ge K. Identification of JmjC domain-containing UTX and JMJD3 as histone H3 lysine 27 demethylases. Proc Natl Acad Sci U S A. 2007;104(47):18439–44. doi: 10.1073/pnas.0707292104 18003914 PMC2141795

[13] Barski A, Cuddapah S, Cui K, Roh TY, Schones DE, Wang Z, et al. High-Resolution Profiling of Histone Methylations in the Human Genome. Cell. 2007;129(4):823–37. doi: 10.1016/j.cell.2007.05.009 17512414

[14] Ferrari KJ, Scelfo A, Jammula SG, Cuomo A, Barozzi I, Stützer A, et al. Polycomb-Dependent H3K27me1 and H3K27me2 Regulate Active Transcription and Enhancer Fidelity. Mol Cell. 2014;53(1):49–62. doi: 10.1016/j.molcel.2013.10.030 24289921

[15] Greenfield A, Carrel L, Pennisi D, Philippe C, Quaderi N, Siggers P, et al. The UTX gene escapes X inactivation in mice and humans. Hum Mol Genet. 1998;7(4):737–42. doi: 10.1093/hmg/7.4.737 9499428

[16] Lan F, Bayliss PE, Rinn JL, Whetstine JR, Wang JK, Chen S, et al. A histone H3 lysine 27 demethylase regulates animal posterior development. Nature. 2007;449(7163):689–94. doi: 10.1038/nature06192 17851529

[17] Faundes V, Goh S, Akilapa R, Bezuidenhout H, Bjornsson HT, Bradley L, et al. Clinical delineation, sex differences, and genotype–phenotype correlation in pathogenic KDM6A variants causing X-linked Kabuki syndrome type 2. Genet Med. Elsevier Masson SAS; 2021;23(7):1202–10. doi: 10.1038/s41436-021-01119-8 33674768 PMC8257478

[18] Luperchio TR, Boukas L, Zhang L, Pilarowski G, Jiang J, Kalinousky A, et al. Leveraging the Mendelian disorders of the epigenetic machinery to systematically map functional epigenetic variation. Elife. 2021 Aug 31;10:1–41. doi: 10.7554/eLife.65884 34463256 PMC8443249

[19] Schott DA, Blok MJ, Gerver WJM, Devriendt K, Zimmermann LJI, Stumpel CTRM. Growth pattern in Kabuki syndrome with a KMT2D mutation. Am J Med Genet Part A. 2016;170(12):3172–9. 27530205 10.1002/ajmg.a.37930

[20] Lindgren AM, Hoyos T, Talkowski ME, Hanscom C, Blumenthal I, Chiang C, et al. Haploinsufficiency of KDM6A is associated with severe psychomotor retardation, global growth restriction, seizures and cleft palate. Hum Genet. 2013;132(5):537–52. doi: 10.1007/s00439-013-1263-x 23354975 PMC3627823

[21] Banka S, Lederer D, Benoit V, Jenkins E, Howard E, Bunstone S, et al. Novel KDM6A (UTX) mutations and a clinical and molecular review of the X-linked Kabuki syndrome (KS2). Clin Genet. 2015;87(3):252–8. doi: 10.1111/cge.12363 24527667

[22] Fahrner JA, Lin WY, Riddle RC, Boukas L, DeLeon VB, Chopra S, et al. Precocious chondrocyte differentiation disrupts skeletal growth in Kabuki syndrome mice. JCI Insight. 2019;4(20). doi: 10.1172/jci.insight.129380 31557133 PMC6824315

[23] Skarnes WC, Rosen B, West AP, Koutsourakis M, Bushell W, Iyer V, et al. A conditional knockout resource for the genome-wide study of mouse gene function. Nature. Nature Publishing Group; 2011;474(7351):337–44. doi: 10.1038/nature10163 21677750 PMC3572410

[24] Wang C, Lee JE, Cho YW, Xiao Y, Jin Q, Liu C, et al. UTX regulates mesoderm differentiation of embryonic stem cells independent of H3K27 demethylase activity. Proc Natl Acad Sci U S A. 2012;109(38):15324–9. doi: 10.1073/pnas.1204166109 22949634 PMC3458330

[25] Sun MMG, Beier F. Chondrocyte hypertrophy in skeletal development, growth, and disease. Birth Defects Res Part C—Embryo Today Rev. 2014;102(1):74–82. doi: 10.1002/bdrc.21062 24677724

[26] Atsumi T, Ikawa Y, Miwa Y, Kimata K. A chondrogenic cell line derived from a differentiating culture of AT805 teratocarcinoma cells. Cell Differ Dev. 1990;30(2):109–16. doi: 10.1016/0922-3371(90)90079-c 2201423

[27] Newton PT, Staines KA, Spevak L, Boskey AL, Teixeira CC, Macrae VE, et al. Chondrogenic ATDC5 cells: An optimised model for rapid and physiological matrix mineralisation. Int J Mol Med. 2012;30(5):1187–93. doi: 10.3892/ijmm.2012.1114 22941229 PMC3573767

[28] Yao Y, Wang Y. ATDC5: An excellent in vitro model cell line for skeletal development. J Cell Biochem. 2013 Jun;114(6):1223–9. doi: 10.1002/jcb.24467 23192741

[29] Myers RB, Fredenburgh JL, Grizzle WE. Carbohydrates. In: Bancroft JD, Gamble M, editors. Theory and Practice of Histological Techniques. 6th Ed. Elsevier; 2008. p. 161–86.

[30] Shen G. The role of type X collagen in facilitating and regulating endochondral ossification of articular cartilage. Orthod Craniofacial Res. 2005;8(1):11–7. doi: 10.1111/j.1601-6343.2004.00308.x 15667640

[31] Klüppel M, Wight TN, Chan C, Hinek A, Wrana JL. Maintenance of chondroitin sulfation balance by chondroitin-4-sulfotransferase 1 is required for chondrocyte development and growth factor signaling during cartilage morphogenesis. Development. 2005;132(17):3989–4003. doi: 10.1242/dev.01948 16079159

[32] Smits P, Dy P, Mitra S, Lefebvre V. Sox5 and Sox6 are needed to develop and maintain source, columnar, and hypertrophic chondrocytes in the cartilage growth plate. J Cell Biol. 2004;164(5):747–58. doi: 10.1083/jcb.200312045 14993235 PMC2172159

[33] Tachibana N, Chijimatsu R, Okada H, Oichi T, Taniguchi Y, Maenohara Y, et al. RSPO2 defines a distinct undifferentiated progenitor in the tendon/ligament and suppresses ectopic ossification. Sci Adv. 2022;8(33):1–15. doi: 10.1126/sciadv.abn2138 35984875 PMC9390986

[34] Tanaka KI, Matsumoto E, Higashimaki Y, Katagiri T, Sugimoto T, Seino S, et al. Role of osteoglycin in the linkage between muscle and bone. J Biol Chem. 2012 ASBMB. Currently published by Elsevier Inc; originally published by American Society for Biochemistry and Molecular Biology.; 2012;287(15):11616–28. doi: 10.1074/jbc.M111.292193 22351757 PMC3320911

[35] Au M, Liu Z, Rong L, Zheng Y, Wen C. Endothelin-1 induces chondrocyte senescence and cartilage damage via endothelin receptor type B in a post-traumatic osteoarthritis mouse model. Osteoarthr Cartil. 2020;28(12):1559–71. 10.1016/j.joca.2020.08.006 32858189

[36] Fitzgerald J, Rich C, Zhou FH, Hansen U. Three novel collagen VI chains, α4(VI), α5(VI), and α6(VI). J Biol Chem. 2008 ASBMB. Currently published by Elsevier Inc; originally published by American Society for Biochemistry and Molecular Biology.; 2008;283(29):20170–80. 10.1074/jbc.M710139200 18400749

[37] Chung HJ, Uitto J. Type VII Collagen: The Anchoring Fibril Protein at Fault in Dystrophic Epidermolysis Bullosa. Dermatol Clin. 2010;28(1):93–105. doi: 10.1016/j.det.2009.10.011 19945621 PMC2791403

[38] Bretaud S, Guillon E, Karppinen SM, Pihlajaniemi T, Ruggiero F. Collagen XV, a multifaceted multiplexin present across tissues and species. Matrix Biol Plus. The Author(s); 2020;6–7:100023. 10.1016/j.mbplus.2020.100023 PMC7852327 33543021

[39] Hopfer U, Fukai N, Hopfer H, Wolf G, Joyce N, Li E, et al. Targeted disruption of Col8a1 and Col8a2 genes in mice leads to anterior segment abnormalities in the eye. FASEB J. 2005;19(10):1232–44. doi: 10.1096/fj.04-3019com 16051690

[40] Kozhemyakina E, Lassar AB, Zelzer E. A pathway to bone: Signaling molecules and transcription factors involved in chondrocyte development and maturation. Dev. 2015;142(5):817–31. doi: 10.1242/dev.105536 25715393 PMC4352987

[41] Shpargel KB, Sengoku T, Yokoyama S, Magnuson T. UTX and UTY Demonstrate Histone Demethylase-Independent Function in Mouse Embryonic Development. PLoS Genet. 2012;8(9). doi: 10.1371/journal.pgen.1002964 23028370 PMC3459986

[42] Van Montfort L, Gerver WJM, Kooger BLS, Plat J, Bierau J, Stumpel CTRM, et al. Follow-Up Study of Growth Hormone Therapy in Children with Kabuki Syndrome: Two-Year Treatment Results. Horm Res Paediatr. 2021;94(7–8):285–96. doi: 10.1159/000519963 34607328

[43] Racine HL, Serrat MA. The Actions of IGF-1 in the Growth Plate and Its Role in Postnatal Bone Elongation. Curr Osteoporos Rep. Current Osteoporosis Reports; 2020;18(3):210–27. doi: 10.1007/s11914-020-00570-x 32415542 PMC7299241

[44] Hallett SA, Ono W, Ono N. The hypertrophic chondrocyte: To be or not to be. Histol Histopathol. 2021;36(10):1021–36. doi: 10.14670/HH-18-355 34137454 PMC8678381

[45] Nilsson A, Carlsson B, Isgaard J, Isaksson OGP, Rymo L. Regulation by GH of insulin-like growth factor-I mRNA expression in rat epiphyseal growth plate as studied with in-situ hybridization. J Endocrinol. 1990;125(1):67–74. doi: 10.1677/joe.0.1250067 2187051

[46] Wu S, Yang W, De Luca F. Insulin-like growth factor-independent effects of growth hormone on growth plate chondrogenesis and longitudinal bone growth. Endocrinol (United States). 2015;156(7):2541–51. doi: 10.1210/en.2014-1983 25910049

[47] Butcher DT, Cytrynbaum C, Turinsky AL, Siu MT, Inbar-Feigenberg M, Mendoza-Londono R, et al. CHARGE and Kabuki Syndromes: Gene-Specific DNA Methylation Signatures Identify Epigenetic Mechanisms Linking These Clinically Overlapping Conditions. Am J Hum Genet. ElsevierCompany.; 2017;100(5):773–88. doi: 10.1016/j.ajhg.2017.04.004 28475860 PMC5420353

[48] Aref-Eshghi E, Bourque DK, Kerkhof J, Carere DA, Ainsworth P, Sadikovic B, et al. Genome-wide DNA methylation and RNA analyses enable reclassification of two variants of uncertain significance in a patient with clinical Kabuki syndrome. Hum Mutat. 2019;40(10):1684–9. doi: 10.1002/humu.23833 31268616

[49] Sadikovic B, Levy MA, Kerkhof J, Aref-eshghi E, Schenkel L, Stuart A, et al. Clinical epigenomics: genome-wide DNA methylation analysis for the diagnosis of Mendelian disorders. Genet Med. Springer US; 2021;1–10. 10.1038/s41436-020-01096-4 PMC8187150 33547396

[50] Aref-Eshghi E, Schenkel LC, Lin H, Skinner C, Ainsworth P, Paré G, et al. The defining DNA methylation signature of Kabuki syndrome enables functional assessment of genetic variants of unknown clinical significance. Epigenetics. 2017;12(11):923–33. doi: 10.1080/15592294.2017.1381807 28933623 PMC5788422

[51] Marwaha A, Costain G, Cytrynbaum C, Mendoza-Londono R, Chad L, Awamleh Z, et al. The utility of DNA methylation signatures in directing genome sequencing workflow: Kabuki syndrome and CDK13-related disorder. Am J Med Genet Part A. 2022;188(5):1368–75. doi: 10.1002/ajmg.a.62650 35043535 PMC9303780

[52] Cobb J, Dierich A, Huss-Garcia Y, Duboule D. A mouse model for human short-stature syndromes identifies Shox2 as an upstream regulator of Runx2 during long-bone development. Proc Natl Acad Sci U S A. 2006;103(12):4511–5. doi: 10.1073/pnas.0510544103 16537395 PMC1450202

[53] Bobick BE, Cobb J. Shox2 regulates progression through chondrogenesis in the mouse proximal limb. J Cell Sci. 2012;125(24):6071–83. doi: 10.1242/jcs.111997 23038774

[54] Wright E, Hargrave MR, Christiansen J, Cooper L, Kun J, Evans T, et al. The Sry-related gene Sox9 is expressed during chondrogenesis in mouse embryos. Nat Genet. 1995 Jan;9(1):15–20. doi: 10.1038/ng0195-15 7704017

[55] Akiyama H, Chaboissier MC, Martin JF, Schedl A, De Crombrugghe B. The transcription factor Sox9 has essential roles in successive steps of the chondrocyte differentiation pathway and is required for expression of Sox5 and Sox6. Genes Dev. 2002;16(21):2813–28. doi: 10.1101/gad.1017802 12414734 PMC187468

[56] Pannier S, Couloigner V, Messaddeq N, Elmaleh-Bergès M, Munnich A, Romand R, et al. Activating Fgfr3 Y367C mutation causes hearing loss and inner ear defect in a mouse model of chondrodysplasia. Biochim Biophys Acta—Mol Basis Dis. Elsevier B.V.; 2009;1792(2):140–7. 10.1016/j.bbadis.2008.11.010 19073250

[57] Ye L, Mishina Y, Chen D, Huang H, Dallas SL, Dallas MR, et al. Dmp1-deficient mice display severe defects in cartilage formation responsible for a chondrodysplasia-like phenotype. J Biol Chem. 2005 ASBMB. Currently published by Elsevier Inc; originally published by American Society for Biochemistry and Molecular Biology.; 2005;280(7):6197–203. doi: 10.1074/jbc.M412911200 15590631 PMC2647591

[58] Carosso GA, Boukas L, Augustin JJ, Nguyen HN, Winer BL, Cannon GH, et al. Precocious neuronal differentiation and disrupted oxygen responses in Kabuki syndrome. JCI Insight. 2019;4(20). doi: 10.1172/jci.insight.129375 31465303 PMC6824316

[59] Gao CW, Lin W, Riddle RC, Kushwaha P, Boukas L, Björnsson HT, et al. Novel mouse model of Weaver syndrome displays overgrowth and excess osteogenesis reversible with KDM6A/6B inhibition. bioRxiv. 2023 Jun 30;1–30. 10.1172/jci.insight.173392 PMC10906465 38015625

[60] Tollervey JR, Lunyak V V. Judge, jury and executioner of stem cell fate. Epigenetics. 2012;7(8):823–40. 22805743 10.4161/epi.21141 PMC3427278

[61] Mackie EJ, Ahmed YA, Tatarczuch L, Chen KS, Mirams M. Endochondral ossification: How cartilage is converted into bone in the developing skeleton. Int J Biochem Cell Biol. 2008;40(1):46–62. doi: 10.1016/j.biocel.2007.06.009 17659995

[62] Aghajanian P, Mohan S. The art of building bone: Emerging role of chondrocyte-to-osteoblast transdifferentiation in endochondral ossification. Bone Res. Springer US; 2018;6(1). doi: 10.1038/s41413-018-0021-z 29928541 PMC6002476

[63] Ono N, Ono W, Nagasawa T, Kronenberg HM. A subset of chondrogenic cells provides early mesenchymal progenitors in growing bones. Nat Cell Biol. 2014 Dec;16(12):1157–67. doi: 10.1038/ncb3067 25419849 PMC4250334

[64] Yang L, Tsang KY, Tang HC, Chan D, Cheah KSE. Hypertrophic chondrocytes can become osteoblasts and osteocytes in endochondral bone formation. Proc Natl Acad Sci U S A. 2014;111(33):12097–102. doi: 10.1073/pnas.1302703111 25092332 PMC4143064

[65] Zhou X, von der Mark K, Henry S, Norton W, Adams H, de Crombrugghe B. Chondrocytes Transdifferentiate into Osteoblasts in Endochondral Bone during Development, Postnatal Growth and Fracture Healing in Mice. PLoS Genet. 2014;10(12). doi: 10.1371/journal.pgen.1004820 25474590 PMC4256265

[66] Hu DP, Ferro F, Yang F, Taylor AJ, Chang W, Miclau T, et al. Cartilage to bone transformation during fracture healing is coordinated by the invading vasculature and induction of the core pluripotency genes. Dev. 2017;144(2):221–34. doi: 10.1242/dev.130807 28096214 PMC5394763

[67] Xing W, Cheng S, Wergedal J, Mohan S. Epiphyseal chondrocyte secondary ossification centers require thyroid hormone activation of Indian hedgehog and osterix signaling. J Bone Miner Res. 2014;29(10):2262–75. doi: 10.1002/jbmr.2256 24753031 PMC4487616

[68] Jing Y, Zhou X, Han X, Jing J, Von Der Mark K, Wang J, et al. Chondrocytes directly transform into bone cells in mandibular condyle growth. J Dent Res. 2015;94(12):1668–75. doi: 10.1177/0022034515598135 26341973 PMC4681473

[69] Aghajanian P, Xing W, Cheng S, Mohan S. Epiphyseal bone formation occurs via thyroid hormone regulation of chondrocyte to osteoblast transdifferentiation. Sci Rep. Springer US; 2017;7(1):1–12. doi: 10.1038/s41598-017-11050-1 28874841 PMC5585223

[70] Lee NK, Sowa H, Hinoi E, Ferron M, Ahn JD, Confavreux C, et al. Endocrine Regulation of Energy Metabolism by the Skeleton. Cell. 2007;130(3):456–69. doi: 10.1016/j.cell.2007.05.047 17693256 PMC2013746

[71] Bjornsson HT, Benjamin JS, Zhang L, Weissman J, Gerber EE, Chen YC, et al. Histone deacetylase inhibition rescues structural and functional brain deficits in a mouse model of Kabuki syndrome. Sci Transl Med. 2014;6(256):1–10. 10.1126/scitranslmed.3009278 PMC4406328 25273096

[72] Cenik BK, Shilatifard A. COMPASS and SWI/SNF complexes in development and disease. Nat Rev Genet. Springer US; 2021;22(1):38–58. doi: 10.1038/s41576-020-0278-0 32958894

[73] Jang Y, Wang C, Zhuang L, Liu C, Ge K. H3K4 Methyltransferase Activity Is Required for MLL4 Protein Stability. J Mol Biol. Elsevier Ltd; 2017;429(13):2046–54. doi: 10.1016/j.jmb.2016.12.016 28013028 PMC5474351

[74] Husmann D, Gozani O. Histone lysine methyltransferases in biology and disease. Nat Struct Mol Biol. Springer US; 2019;26(10):880–9. doi: 10.1038/s41594-019-0298-7 31582846 PMC6951022

[75] Wang SP, Tang Z, Chen CW, Shimada M, Koche RP, Wang LH, et al. A UTX-MLL4-p300 Transcriptional Regulatory Network Coordinately Shapes Active Enhancer Landscapes for Eliciting Transcription. Mol Cell. Elsevier Inc.; 2017;67(2):308–321.e6. doi: 10.1016/j.molcel.2017.06.028 28732206 PMC5574165

[76] Morales Torres C, Laugesen A, Helin K. Utx Is Required for Proper Induction of Ectoderm and Mesoderm during Differentiation of Embryonic Stem Cells. PLoS One. 2013;8(4):1–15. doi: 10.1371/journal.pone.0060020 23573229 PMC3616089

[77] Benjamin JS, Pilarowski GO, Carosso GA, Zhang L, Huso DL, Goff LA, et al. A ketogenic diet rescues hippocampal memory defects in a mouse model of Kabuki syndrome. Proc Natl Acad Sci U S A. 2017 Jan 3;114(1):125–30. doi: 10.1073/pnas.1611431114 27999180 PMC5224378

[78] Zhang L, Pilarowski G, Pich EM, Nakatani A, Dunlop J, Baba R, et al. Inhibition of KDM1A activity restores adult neurogenesis and improves hippocampal memory in a mouse model of Kabuki syndrome. Mol Ther—Methods Clin Dev. Elsevier Ltd.; 2021;20(March):779–91. 10.1016/j.omtm.2021.02.011 PMC7940709 33738331

[79] Bouxsein ML, Boyd SK, Christiansen BA, Guldberg RE, Jepsen KJ, Müller R. Guidelines for assessment of bone microstructure in rodents using micro-computed tomography. J Bone Miner Res. 2010;25(7):1468–86. doi: 10.1002/jbmr.141 20533309

[80] Leong ASY, Haffajee Z. Citraconic anhydride: A new antigen retrieval solution. Pathology. 2010;42(1):77–81. doi: 10.3109/00313020903434439 20025485

[81] Patro R, Duggal G, Love MI, Irizarry RA, Kingsford C. Salmon provides fast and bias-aware quantification of transcript expression. Nat Methods. Nature Publishing Group; 2017;14(4):417–9. doi: 10.1038/nmeth.4197 28263959 PMC5600148

[82] Love MI, Soneson C, Hickey PF, Johnson LK, Tessa Pierce N, Shepherd L, et al. Tximeta: Reference sequence checksums for provenance identification in RNA-seq. PLoS Comput Biol. 2020;16(2):1–13. doi: 10.1371/journal.pcbi.1007664 32097405 PMC7059966

[83] Leek JT, Johnson WE, Parker HS, Jaffe AE, Storey JD. The SVA package for removing batch effects and other unwanted variation in high-throughput experiments. Bioinformatics. 2012;28(6):882–3. doi: 10.1093/bioinformatics/bts034 22257669 PMC3307112

[84] Love MI, Huber W, Anders S. Moderated estimation of fold change and dispersion for RNA-seq data with DESeq2. Genome Biol. 2014;15(12):1–21. doi: 10.1186/s13059-014-0550-8 25516281 PMC4302049

[85] Durinck S, Moreau Y, Kasprzyk A, Davis S, De Moor B, Brazma A, et al. BioMart and Bioconductor: A powerful link between biological databases and microarray data analysis. Bioinformatics. 2005;21(16):3439–40. doi: 10.1093/bioinformatics/bti525 16082012

[86] Edgar R, Domrachev M, Lash AE. Gene Expression Omnibus: NCBI gene expression and hybridization array data repository. Nucleic Acids Res. 2002;30(1):207–10. doi: 10.1093/nar/30.1.207 11752295 PMC99122

