# Supplementary material for: Growth deficiency in a mouse model of Kabuki syndrome 2 bears mechanistic similarities to Kabuki syndrome 1
Source: PLoS Genet. 2024 Jun 10;20(6):e1011310. doi: 10.1371/journal.pgen.1011310 (PMC11192384; doi:10.1371/journal.pgen.1011310)
Supplement: S1 Fig — (PDF) [file pgen.1011310.s001.pdf]

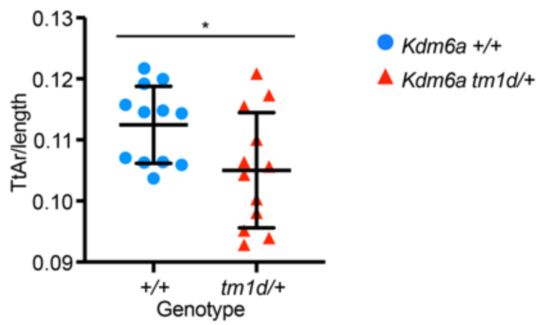

S1 Fig. Tissue area of *Kdm6a*$^{tm1d/+}$ and *Kdm6a*$^{+/+}$ littermates, normalized by femur length. *Kdm6a*$^{tm1d/+}$ mice have decreased tissue area compared to *Kdm6a*$^{+/+}$ even after adjustment for shorter femur lengths. *Kdm6a*$^{+/+}$ n=12; *Kdm6a*$^{tm1d/+}$ n=12. Blue circles: *Kdm6a*$^{+/+}$, red triangles: *Kdm6a*$^{tm1d/+}$. *p < 0.05, two-tailed unpaired Student's t-test. All error bars represent mean ± 1 SD. TtAr, total area.
