# Supplementary material for: Growth deficiency in a mouse model of Kabuki syndrome 2 bears mechanistic similarities to Kabuki syndrome 1
Source: PLoS Genet. 2024 Jun 10;20(6):e1011310. doi: 10.1371/journal.pgen.1011310 (PMC11192384; doi:10.1371/journal.pgen.1011310)
Supplement: S2 Fig — (PDF) [file pgen.1011310.s002.pdf]

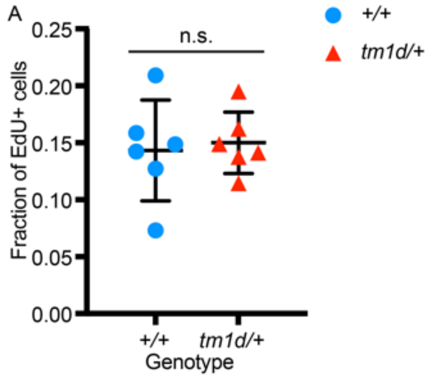

S2 Fig. Cell proliferation rate does not differ between growth plates from *Kdm6a*$^{tm1d/+}$ and *Kdm6a*$^{+/+}$ mice. Calculated fraction of EdU+ cells in the growth plates of *Kdm6a*$^{tm1d/+}$ mice and *Kdm6a*$^{+/+}$ littermate controls. Blue circles: *Kdm6a*$^{+/+}$, red triangles: *Kdm6a*$^{tm1d/+}$. n.s., non-significant, two-tailed unpaired Student's t-test. All error bars represent mean ± 1 SD.
