# Supplementary material for: Growth deficiency in a mouse model of Kabuki syndrome 2 bears mechanistic similarities to Kabuki syndrome 1
Source: PLoS Genet. 2024 Jun 10;20(6):e1011310. doi: 10.1371/journal.pgen.1011310 (PMC11192384; doi:10.1371/journal.pgen.1011310)
Supplement: S3 Fig — (PDF) [file pgen.1011310.s003.pdf]

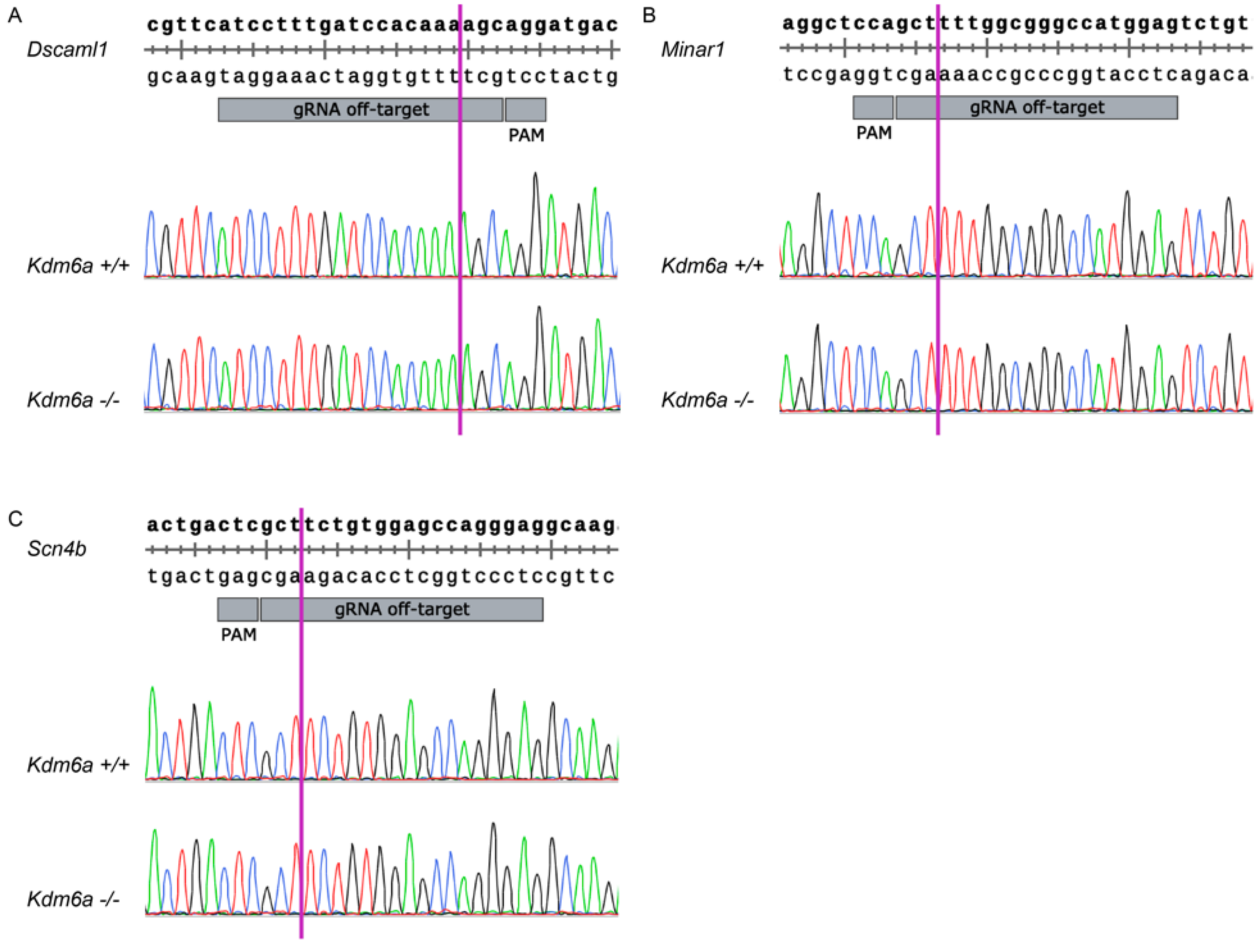


**S3 Fig. Absence of off-target editing in *Kdm6a*$^{+/+}$ and *Kdm6a*$^{-/-}$ cell lines at top predicted exonic sites.** Representative chromatogram traces from *Kdm6a*$^{+/+}$ and *Kdm6a*$^{-/-}$ lines are shown for: (A) *Dscaml1*, (B) *Minar1*, and (C) *Scn4b*. Purple line indicates predicted off-target Cas9 cleavage site.
