# Supplementary material for: Growth deficiency in a mouse model of Kabuki syndrome 2 bears mechanistic similarities to Kabuki syndrome 1
Source: PLoS Genet. 2024 Jun 10;20(6):e1011310. doi: 10.1371/journal.pgen.1011310 (PMC11192384; doi:10.1371/journal.pgen.1011310)
Supplement: S4 Fig — (PDF) [file pgen.1011310.s004.pdf]

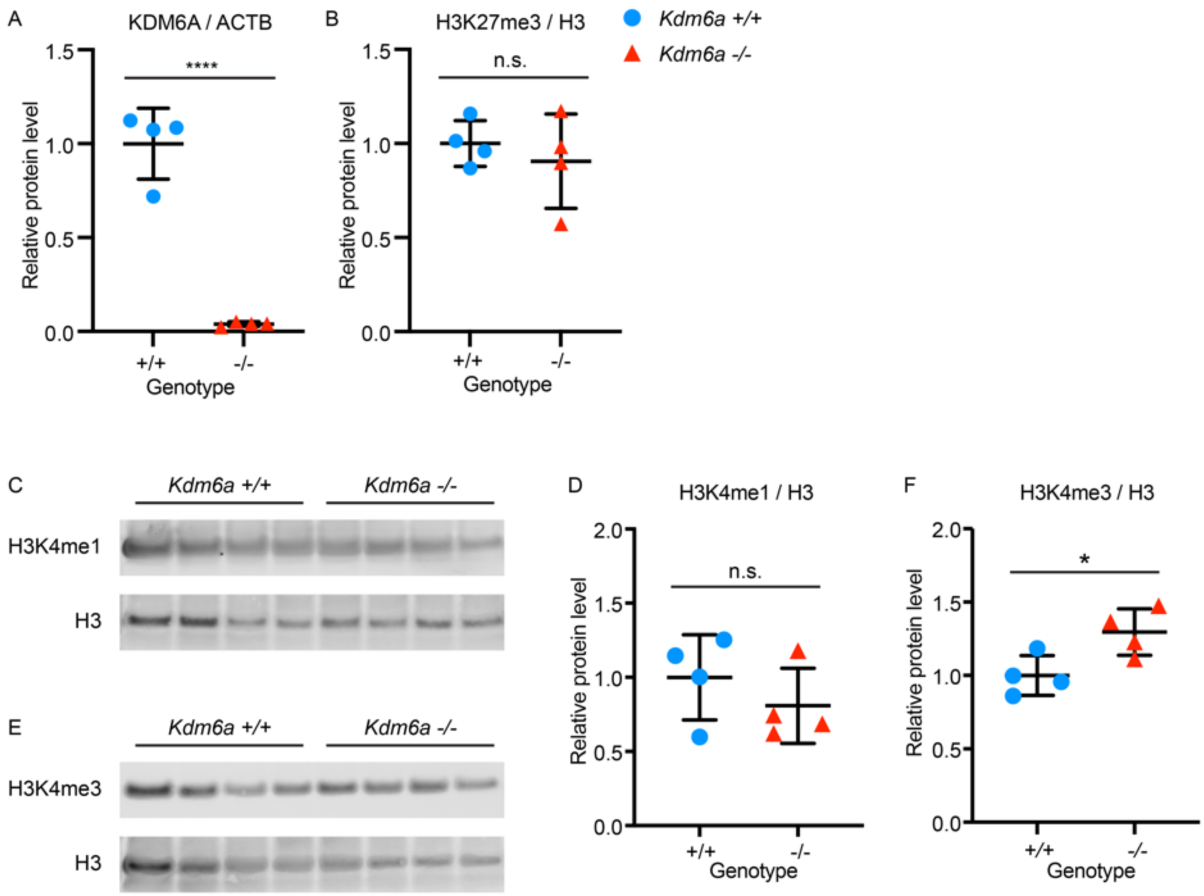

S4 Fig. *Kdm6a*$^{-/-}$ cells do not express KDM6A and have increased global H3K4me3. For all densitometry panels, a relative protein level of 1.0 corresponds to the mean densitometry of *Kdm6a*$^{+/+}$ cell lines. (A) *Kdm6a*$^{-/-}$ cells have negligible KDM6A expression, normalized against ACTB loading controls. (B) There is no significant difference in H3K27me3 levels between *Kdm6a*$^{-/-}$ and *Kdm6a*$^{+/+}$ cell lines, with H3 serving as loading control. (C) Western blot and (D) densitometry quantification show no difference in global H3K4me1 between *Kdm6a*$^{-/-}$ and *Kdm6a*$^{+/+}$ cell lines. H3K4me3 is significantly increased in *Kdm6a*$^{-/-}$ cells by (E) western blot and (F) densitometry quantification. Blue circles: *Kdm6a*$^{+/+}$, red triangles: *Kdm6a*$^{-/-}$. *$p < 0.05$, ****$p < 0.0001$, two-tailed unpaired Student's t-test. n.s., non-significant. All error bars represent mean ± 1 SD.
