# Supplementary material for: Growth deficiency in a mouse model of Kabuki syndrome 2 bears mechanistic similarities to Kabuki syndrome 1
Source: PLoS Genet. 2024 Jun 10;20(6):e1011310. doi: 10.1371/journal.pgen.1011310 (PMC11192384; doi:10.1371/journal.pgen.1011310)
Supplement: S5 Fig — (PDF) [file pgen.1011310.s005.pdf]

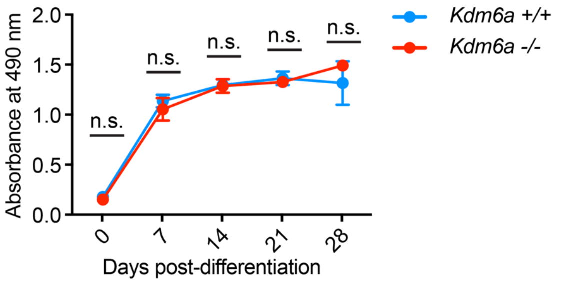


**S5 Fig. *Kdm6a*$^{-/-}$ cells do not differ in proliferation rate from *Kdm6a*$^{+/+}$.** The MTT assay was performed. Colorimetric conversion of a tetrazolium compound to formazan, as measured by absorbance at 490 nm, is directly proportional to the number of live cells in culture. The mean of four cell lines per genotype is displayed. Blue circles: *Kdm6a*$^{+/+}$, red circles: *Kdm6a*$^{-/-}$. Two-tailed unpaired Student's t-test. n.s., non-significant. All error bars represent mean ± 1 SD.
