# Supplementary material for: Growth deficiency in a mouse model of Kabuki syndrome 2 bears mechanistic similarities to Kabuki syndrome 1
Source: PLoS Genet. 2024 Jun 10;20(6):e1011310. doi: 10.1371/journal.pgen.1011310 (PMC11192384; doi:10.1371/journal.pgen.1011310)
Supplement: S6 Fig — (PDF) [file pgen.1011310.s006.pdf]

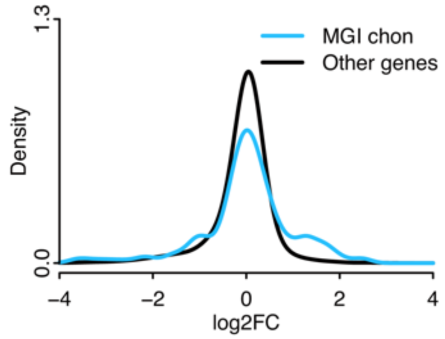

S6 Fig. Density distribution of expression fold-changes comparing *Kdm6a*$^{-/-}$ to *Kdm6a*$^{+/+}$ chondrocytes. A greater proportion of genes annotated by MGI as involved in cartilage development (blue line) exhibit higher absolute fold-changes than non-chondrogenic genes (black line). MGI chon, MGI-annotated chondrogenic genes.
