# Supplementary material for: Growth deficiency in a mouse model of Kabuki syndrome 2 bears mechanistic similarities to Kabuki syndrome 1
Source: PLoS Genet. 2024 Jun 10;20(6):e1011310. doi: 10.1371/journal.pgen.1011310 (PMC11192384; doi:10.1371/journal.pgen.1011310)
Supplement: S7 Fig — (PDF) [file pgen.1011310.s007.pdf]

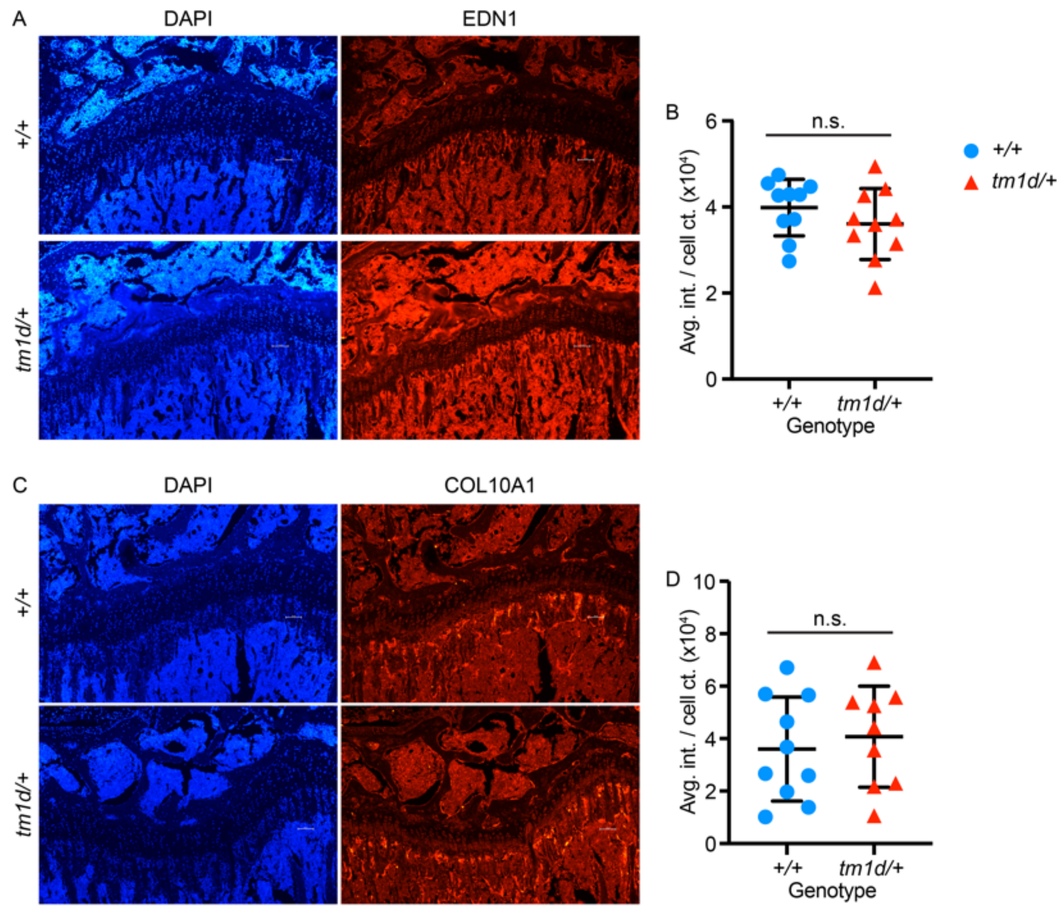

S7 Fig. Growth plates from *Kdm6a*$^{tm1d/+}$ and *Kdm6a*$^{+/+}$ mice do not differ in protein expression of EDN1 and COL10A1. Immunofluorescence was performed on proximal tibial growth plates from *Kdm6a*$^{+/+}$ and *Kdm6a*$^{tm1d/+}$ mice. (A) Representative images of DAPI nuclear staining (left panels) and immunofluorescence for EDN1 (right panels). (B) Quantification of immunofluorescence for EDN1 in the growth plate, normalized by cell count. (C) Representative images of DAPI nuclear staining and immunofluorescence for COL10A1. (D) Quantification of immunofluorescence for COL10A1. Blue circles: *Kdm6a*$^{+/+}$, red triangles: *Kdm6a*$^{tm1d/+}$. All error bars represent mean ± 1 SD. Two-tailed unpaired Student's t-test. n.s., non-significant; avg. int., average intensity; cell ct., cell count.
