# Supplementary material for: Growth deficiency in a mouse model of Kabuki syndrome 2 bears mechanistic similarities to Kabuki syndrome 1
Source: PLoS Genet. 2024 Jun 10;20(6):e1011310. doi: 10.1371/journal.pgen.1011310 (PMC11192384; doi:10.1371/journal.pgen.1011310)
Supplement: S8 Fig — (PDF) [file pgen.1011310.s008.pdf]

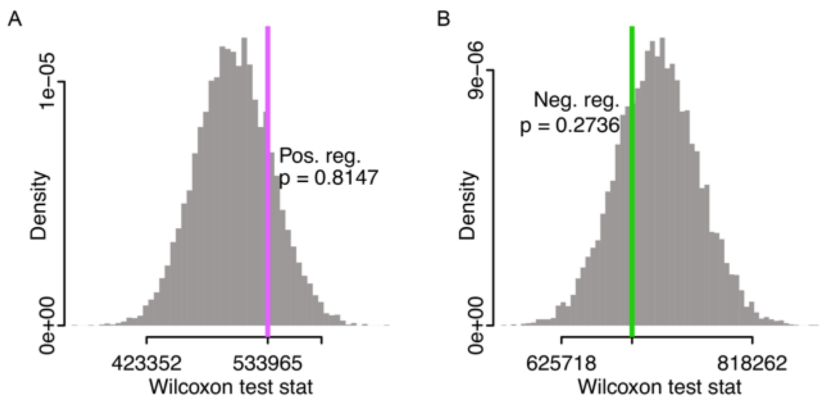

S8 Fig. *Kdm6a*$^{-/-}$ cells do not exhibit collective dysregulation of cell size pathways. Wilcoxon rank-sum test statistic for (A) MGI positive regulators of cell size (magenta line, p=0.8147) and (B) MGI negative regulators of cell size (green line, p=0.2738). The simulated distribution of test statistics is displayed for each (gray). Pos. reg., positive regulators; neg. reg., negative regulators.
