# Supplementary material for: Growth deficiency in a mouse model of Kabuki syndrome 2 bears mechanistic similarities to Kabuki syndrome 1
Source: PLoS Genet. 2024 Jun 10;20(6):e1011310. doi: 10.1371/journal.pgen.1011310 (PMC11192384; doi:10.1371/journal.pgen.1011310)
Supplement: S9 Fig — (PDF) [file pgen.1011310.s009.pdf]

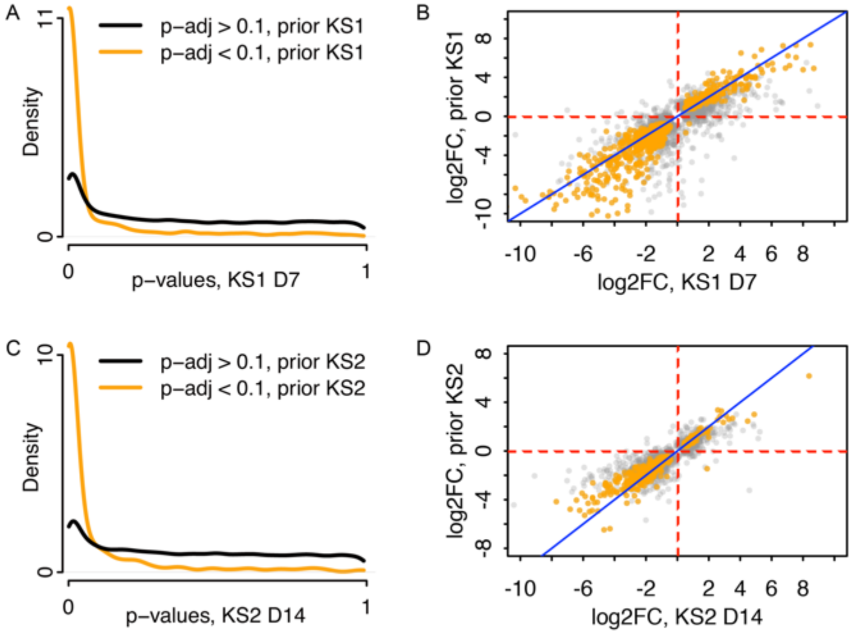

S9 Fig. Validation of KS1 and KS2 RNA-seq data by comparison to prior datasets. (A) Conditional p-value density plot displaying gene-wise p-values from the *Kmt2d*$^{-/-}$ versus *Kmt2d*$^{+/+}$ contrast at Day 7 of chondrogenic differentiation, stratified by significance in a prior dataset comparing *Kmt2d*$^{-/-}$ versus *Kmt2d*$^{+/+}$ cells, also collected at Day 7 (22). Orange: genes that were significant (FDR < 0.1) in the prior dataset; black: non-significant in prior dataset. (B) Scatter plot of gene-wise $\log_2$(fold-changes) in present and prior datasets at Day 7 shows high concordance in fold-change directionalities between datasets. A similar analysis was performed for the *Kdm6a*$^{-/-}$ versus *Kdm6a*$^{+/+}$ contrast at Day 14: (C) conditional p-value histogram and (D) $\log_2$(fold-change) scatter plot.
