# Supplementary material for: Growth deficiency in a mouse model of Kabuki syndrome 2 bears mechanistic similarities to Kabuki syndrome 1
Source: PLoS Genet. 2024 Jun 10;20(6):e1011310. doi: 10.1371/journal.pgen.1011310 (PMC11192384; doi:10.1371/journal.pgen.1011310)
Supplement: S1 Table — Kdm6a-/- frameshift variants generated using CRISPR/Cas9 gene editing. (PDF) [file pgen.1011310.s010.pdf]

S1 Table. ATDC5 cell line genotypes

Line	Genotype	Allele variants
10-37	Compound het	NM_009483.2:c.496del p.(Cys166Valfs*16)<br>NM_009483.2:c.483_493del p.(Asp162Leufs*21)
10-39	Compound het	NM_009483.2:c.495_496del p.(Phe165Leufs*21)<br>NM_009483.2:c.493_494insA p.(Phe165Tyrfs*22)
96-15	Compound het	NM_009483.2:c.495_496del p.(Phe165Leufs*21)<br>NM_009483.2:c.496del p.(Cys166Valfs*16)
96-20	Compound het	NM_009483.2:c.496dup p.(Cys166Leufs*21)<br>NM_009483.2:c.496del p.(Cys166Valfs*16)
96-21	Compound het	NM_009483.2:c.495_496del p.(Phe165Leufs*21)<br>NM_009483.2:c.496dup p.(Cys166Leufs*21)
10-3	Wild-type	
10-29	Wild-type	
96-13	Wild-type	
96-14	Wild-type	
96-19	Wild-type	
