# Supplementary material for: Growth deficiency in a mouse model of Kabuki syndrome 2 bears mechanistic similarities to Kabuki syndrome 1
Source: PLoS Genet. 2024 Jun 10;20(6):e1011310. doi: 10.1371/journal.pgen.1011310 (PMC11192384; doi:10.1371/journal.pgen.1011310)
Supplement: S2 Table — Detailed list of reagents used in the manuscript. (PDF) [file pgen.1011310.s011.pdf]

S2 Table. Reagents

	Species	Manufacturer	Catalog no.	Dilution
anti-H3K27me3	Rabbit	Millipore Sigma	07-449	1:1000 (WB)
anti-H3K27me1	Rabbit	Abcam	ab8895	1:500 (WB)
anti-H3	Rabbit	Cell Signaling Technology	4499	1:2000 (WB)
anti-KDM6A	Rabbit	Cell Signaling Technology	33510	1:1000 (WB)
anti-KMT2D	Rabbit	Millipore Sigma	ABE1867	1:500 (WB)
anti-ACTB	Mouse	Cell Signaling Technology	3700	1:1000 (WB)
anti-Vinculin	Mouse	Millipore Sigma	V9131	1:10,000 (WB)
IRDye 800CW Donkey anti-Rabbit IgG	Donkey	LI-COR	926-32213	1:10,000 (WB)
IRDye 680RD Donkey anti-Mouse IgG	Donkey	LI-COR	926-68072	1:10,000 (WB)
anti-COL10A1	Rabbit	Novus Bio	NBP3-03757	1:100 (IF)
anti-EDN1	Rabbit	Abcam	ab117757	1:2000 (IF)
Alexa Fluor 555 – Conjugated anti-Rabbit	Goat	ThermoFisher Scientific	A21429	1:400 (IF)
